# On Expression Patterns and Developmental Origin of Human Brain Regions

**DOI:** 10.1371/journal.pcbi.1005064

**Published:** 2016-08-26

**Authors:** Lior Kirsch, Gal Chechik

**Affiliations:** The Gonda Multidisciplinary Brain Research Center, Bar-Ilan University, Ramat Gan, Israel; CHB, Harvard Medical School, UNITED STATES

## Abstract

Anatomical substructures of the human brain have characteristic cell-types, connectivity and local circuitry, which are reflected in area-specific transcriptome signatures, but the principles governing area-specific transcription and their relation to brain development are still being studied. In adult rodents, areal transcriptome patterns agree with the embryonic origin of brain regions, but the processes and genes that preserve an embryonic signature in regional expression profiles were not quantified. Furthermore, it is not clear how embryonic-origin signatures of adult-brain expression interplay with changes in expression patterns during development. Here we first quantify which genes have regional expression-patterns related to the developmental origin of brain regions, using genome-wide mRNA expression from post-mortem adult human brains. We find that almost all human genes (92%) exhibit an expression pattern that agrees with developmental brain-region ontology, but that this agreement changes at multiple phases during development. Agreement is particularly strong in neuron-specific genes, but also in genes that are not spatially correlated with neuron-specific or glia-specific markers. Surprisingly, agreement is also stronger in early-evolved genes. We further find that pairs of similar genes having high agreement to developmental region ontology tend to be more strongly correlated or anti-correlated, and that the strength of spatial correlation changes more strongly in gene pairs with stronger embryonic signatures. These results suggest that transcription regulation of most genes in the adult human brain is spatially tuned in a way that changes through life, but in agreement with development-determined brain regions.

## Author Summary

Genome-wide measurements of gene expression across the human brain can reveal new principles of brain organization and function. To achieve this, we aim to discover which genes are differentially expressed and in what brain regions. We found that almost all genes in the adult human brain bear a developmental ‘footprint’ which determines their areal expression-pattern based on the developmental ontology of brain regions, while at the same time their spatial expression pattern changes during life. Furthermore, pairs of paralog genes and similar genes with stronger embryonic footprint, tend to be more strongly correlated (or anti correlated) suggesting that their expression is more strongly spatially tuned, and this tuning changes in development.

## Introduction

The human brain is organized in a hierarchy of multiple substructures, whose cell composition and circuitry are believed to allow each substructure to carry out its distinct function. While physiological and histological differences and similarities between structures have been intensively studied [1–4], the molecular profiles giving rise to those differences are far from being understood. Specifically, it is not known which principles govern the expression patterns of genes across the **adult brain** and what determines their spatial organization. Recent high-resolution genome-wide transcriptome profiling studies allow addressing these questions [5,6]. The current paper explores the role of development in determining adult expression patterns.

**Fig 1:**
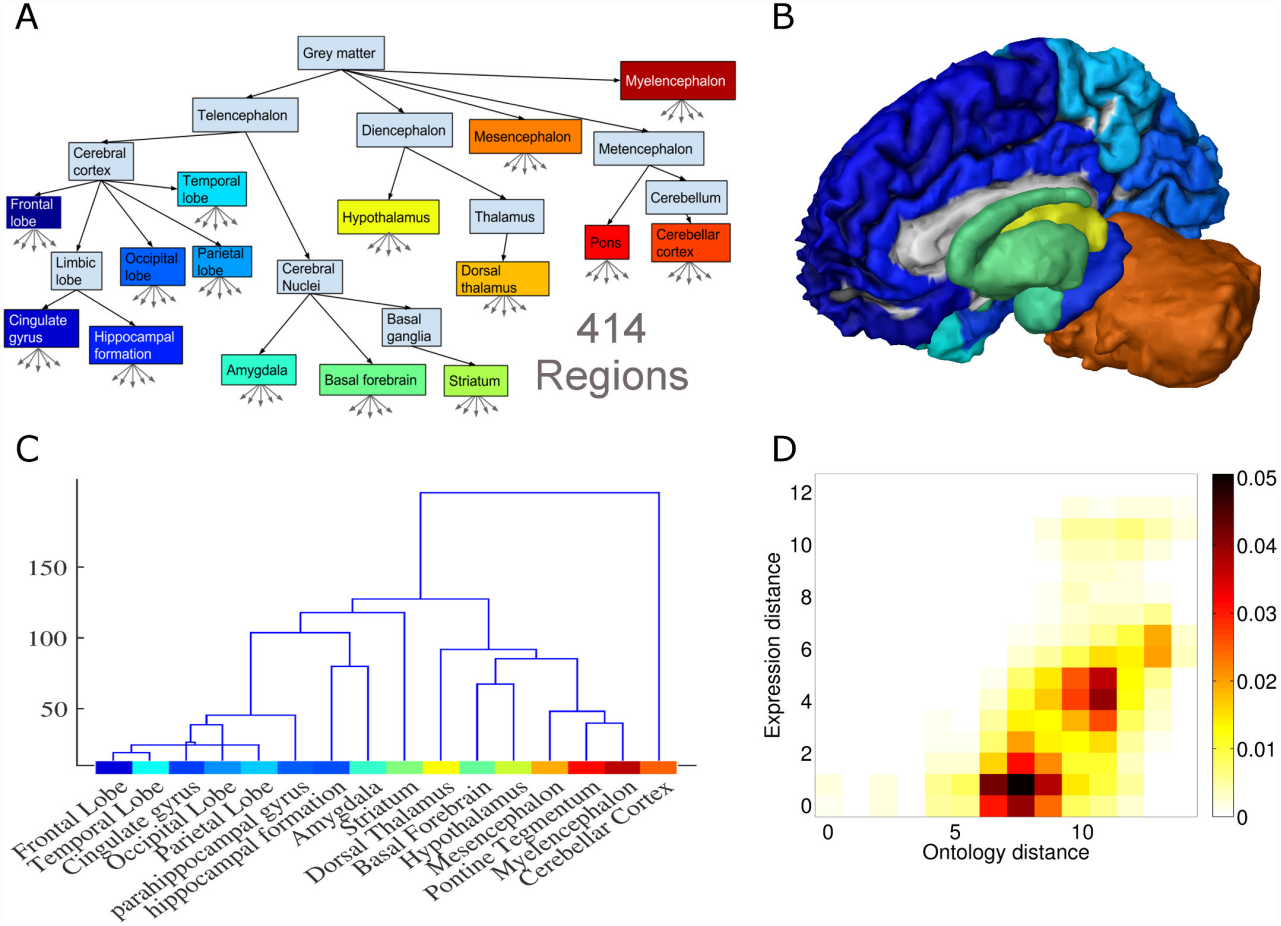
Brain-region ontology and the BRO-agreement score. **(A)** Illustration of the ontology region tree showing 16 brain structures studied. The full ontology contains 1534 regions, not shown. **(B)** A 3D model brain illustrating 16 brain regions using the same colors as in A. The left cortex is not shown in order to expose the inner structures. **(C)** Hierarchical clustering of 16 human brain structures. Agglomerative linking of regions by their average expression profile yields a tree structure that agrees the with ontology tree. The color above the region name matches the colors in the region ontology tree in Fig 1A. **(D)** The joint distribution of expression distances and ontology distances across all pairs of tissue samples, as computed for the gene *NEUROD1*. The two distance measures are strongly correlated (Spearman ρ = 0.65, n = 6.85M, *p*-value < 10^−15^), showing that the spatial expression pattern agrees with the ontology.

In early development of the vertebrate nervous system, the posterior part of the neural tube develops into the spinal cord, and its anterior part divides into three primary vesicles: the prosencephalon, the mesencephalon and the rombencephalon. The prosencephalon further develops into two secondary vesicles: the telencephalon and the diencephalon. The most posterior vesicle, the rombencephalon, forms two secondary vesicles as well, the metencephalon, and the myelencephalon. These five vesicles are aligned along the rostral-caudal axis of the developing brain and establish the primary organization of the central nervous system (Fig 1A) [7].

During early development, several gene families exhibit distinct spatial expression patterns [8], including, for example, genes involved in axon guidance and in segmentation and compartmentalization of brain regions [9,10]. Genes including netrins, semaphorins, and ephrins function as molecular signatures, guiding axons to form long-range connections [11–13]. Patterning genes [14], like the Hox gene family, play a crucial role in forming brain regions [15]. Some genes, including Hox genes, were shown to retain unique expression patterns related to tissue specificity across the adult body [16–18]. Many other genes, change their expression patterns during development, both before or after birth [19–21]. However, for most genes that are not directly involved in brain development, it is usually not known which processes determine their spatial expression pattern across the adult brain.

Zapala and colleagues [22] studied areal expression across the adult mouse brain by clustering brain regions based on the expression levels of ~2000 genes. They found that the pattern of gene expression in a brain region was correlated with the position of that region along the anterior-posterior axis of the neural tube. Regions with similar expression profiles often shared a common embryonic origin. One possible explanation of this finding would be that brain regions sharing an embryonic origin contain similar cell-types, which in turn express shared gene markers. For example, pyramidal neurons are the main excitatory neurons both in the neocortex and in the hippocampus. The agreement between embryonic origin and expression similarity may therefore be explained by a small number of cell-type specific gene markers. Indeed, French *et al*. have shown that regions with similar expression profiles tend to have similar neuronal connectivity patterns [23]. Ko *et al*. have shown that neuron-specific and astrocyte-specific gene markers show distinct patterns of expression across brain regions [24]. Grange *et al*. have estimated the spatial densities of 64 cell types in the mouse brain and identified genes with a localized pattern of expression [25]. The question remains however, how genome-wide is the agreement of expression-pattern with embryonic origin and how does this agreement develop during the lifetime of an organism.

It is also not clear to what extent expression differences are pronounced between sub regions of the major brain structures. Specifically, the cortex is sometimes viewed as largely homogeneous in various properties, including neuronal density, connectivity patterns and distribution of cell-types, properties which were shown to be consistent across cortical sub-regions [4,26]. Also, cortical microcircuits can be induced to perform processing tasks that are naturally performed in other regions [27–30]. These views are in agreement with the results of Ko *et al*. [24], where clustering brain voxels based on their expression profile showed that the cortex is significantly different from other structures, but is largely homogeneous by itself. However, the level of homogeneity of cortex expression patterns has not been quantified directly so far.

To quantify the relation between expression patterns across the adult human brain and the embryonic origin of the corresponding brain regions on a gene-by-gene basis, we analyze two genome-wide mRNA expression datasets. For each gene separately, we computed an index that measures how its expression pattern in the adult agrees with a brain-region ontology based on embryonic brain development. Surprisingly, we found that almost all genes exhibit a spatial expression pattern that significantly agrees with the brain-region ontology. This effect is particularly strong in neuron-specific genes as expected, but also in many genes that serve more generic functions. This suggests that the brain tunes the areal expression pattern of genes in a way that strongly depends on embryonic development, and this holds even for genes that participate in brain-wide functions. Furthermore, pairs of genes sharing related functions tend to be spatially more anti-correlated if their expression pattern agrees with the brain-region ontology.

## Results

To characterize distinct gene expression patterns across the adult human brain, we analyzed genome-wide expression measurements from two sources. First, a set of 3702 samples from 6 adult post-mortem human brains [5], and second, a set of 491 samples from 20 adult post-mortem human brains [6]. See Methods for details on both datasets. In the results below, we refer to these datasets as *ABA6-2013* and *Kang-2011*.

### Spatial pattern of expression and agreement with brain-region ontology

We evaluated how expression of each individual gene across the brain agrees with an ontology of brain regions provided by the Allen Institute (http://human.brain-map.org) [31]. This ontology is coarsely based on brain development, covering both developing and adult human brain structures. The fine structure of the ontology, including cortical parcellation, is based on classical cytoarchitecture. Instead of analyzing expression variability across regions using a ‘flat’ representation of regions, the ontology allows to take into account the ‘structured’ similarities among regions stemming from the shared embryonic origin of regions. We used the full tree ontology which contained 1534 brain regions. Brain samples from the *ABA6-2013* dataset were associated with 414 ontology regions, and those from the *Kang-2011* dataset were associated with 16 regions of the ontology (see Methods S2 and S3 Tables). Fig 1A depicts the region-ontology tree at a coarse resolution for visualization purposes. Nodes in the tree are colored blue-to-red, roughly corresponding to position of regions on the anterior-posterior axis. Fig 1B depicts the same regions on a 3D model of a human brain using the corresponding colors.

In the mouse brain, regions that share similar expression patterns group in a way that matches the region ontology [22,32]. To test if these result are reproduced in human, we clustered brain regions based on the full-genome expression profile (see Methods). The resulting clustering agrees with the findings in the mouse. Fig 1C shows the hierarchical clustering of 16 brain regions from *ABA6-2013*, where brain regions with a common developmental origin, share similar expression patterns. However, this clustering depends on the joint expression patterns of all genes, and the question remains: which genes and processes contribute to this effect?

To quantify how the expression pattern of each individual gene agrees with the region ontology, we defined an index, which we call *Brain-Region Ontology* agreement score (BRO-agreement score), calculated as follows. For a given gene, we consider all pairs of tissue-samples, and for each pair we computed two measures of distances. The first, ***expression distance***, is the absolute difference of expression values in the two samples. The second, ***ontology distance***, is the distance between the corresponding regions in the ontology tree. The BRO score for each gene is defined as the spearman correlation between the two distances computed for all tissue pairs. It provides a measure of the agreement between expression difference and ontology distance (for more details see Methods). We also tested a second index based on triplet ranking which yielded similar results (see Methods). To illustrate the use of the BRO score, consider the expression pattern of the gene *NEUROD1*, a transcription factor involved in regulation of brain development. Fig 1D depicts the joint distribution of the ontology distance and the expression distance computed for *NEUROD1* expression measured at all tissue pairs. The two distance measures are strongly correlated, with a BRO score of 0.65, suggesting that the variability in the expression of *NEUROD1* across the adult brain is largely explained by the position of the region in the development region ontology.

$$BRO\_score(gene\;i\text{):=}spearman\;corr_{(a,b)\in all\;sample\;pairs}(d_{tree}(a,b),\;d_{expression}(a_{i},\;b_{i}))$$

We computed the BRO index for every gene in the *ABA6-2013* dataset based on a fine-resolution ontology of 414 brain regions (see Methods). To assess significance, the BRO score of each gene was compared to a randomized BRO score obtained by permuting the expression profile of the gene across regions. We find that 92% of genes significantly agree with the brain-region ontology more than random (FDR–corrected, *q*-value < 0.01). Using the triplets score (see Methods), 95% of the genes in this dataset were significant. This surprisingly-high fraction suggests that most genes have distinct areal expression patterns across the adult brain, and that these patterns are largely determined by the embryonic origin of the brain regions they are expressed in.

**Fig 2:**
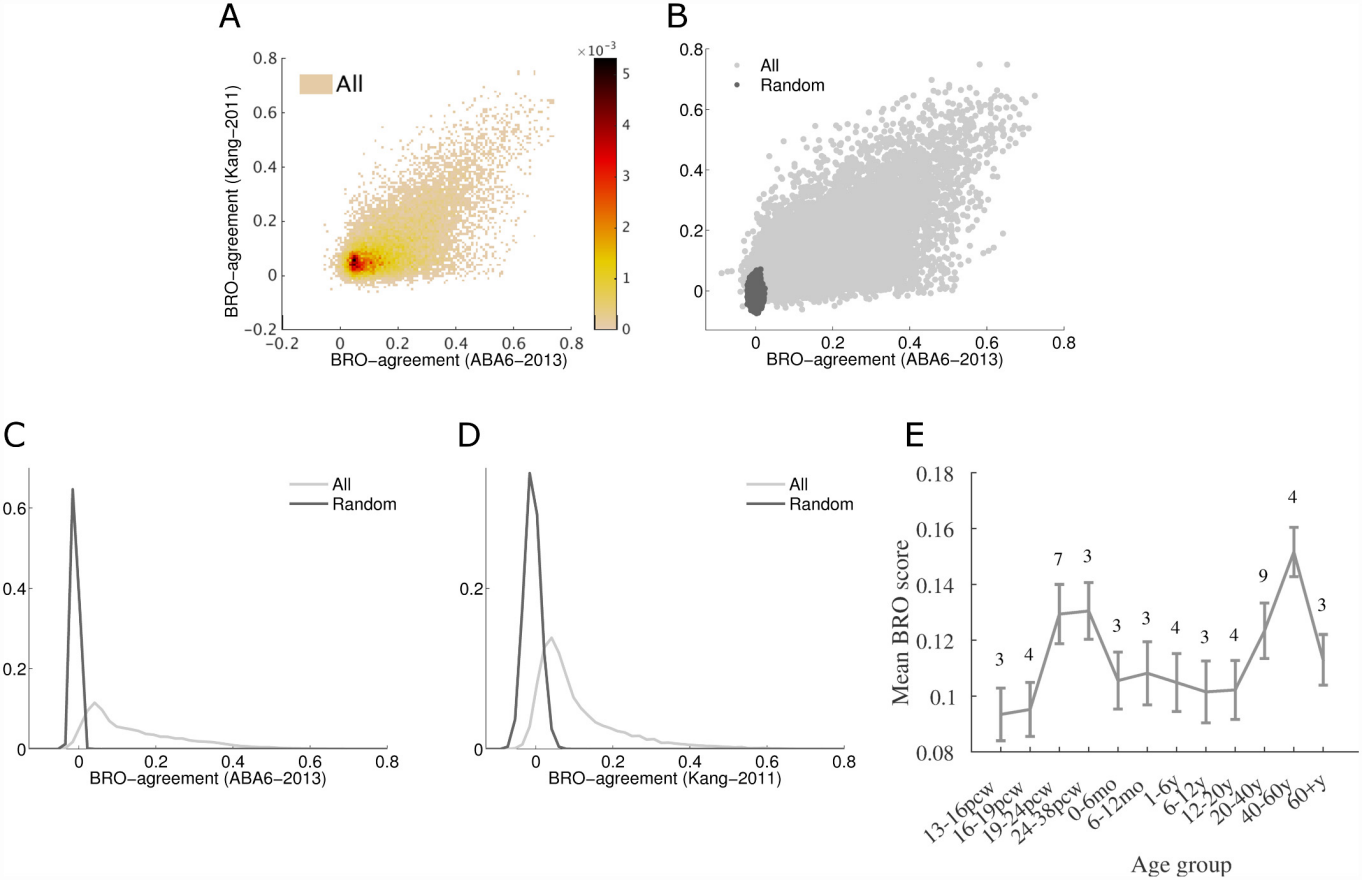
Distribution of BRO-agreement scores of individual genes. **(A)** Heatmap showing the joint distribution of BRO-agreement scores of all genes in 16 regions of *ABA6-2013* (absica) and *Kang-2011* (ordinate). Colors correspond to the density. **(B)** A scatter plot showing BRO-agreement scores for the two datasets in A. Each light-grey dot corresponds to a single gene (a total of 17K genes). Dark-grey dots correspond to permuted data (see Methods). The BRO scores are significantly correlated across the two datasets (Spearman, *ρ* = 0.53, n = 16947, *p*-value<10^−16^). **(C)** Marginal distribution of BRO scores in the *ABA6-2013* dataset. BRO scores for most genes are significantly greater than randomized scores. **(D)** Same as C, for the *Kang-2011* data. **(E)** BRO-agreement scores traced through life based on the full developmental dataset in *Kang-2011*. Samples are aggregated based on the developmental stages defined in [6]. Numbers above the line denote the number of subjects in each age group.

To test reproducibility and robustness of these results, we compare the BRO index obtained for every gene in the two microarray datasets (see Methods), covering a total of 26 postmortem brains. Fig 2A shows the joint distribution of BRO-agreement scores (light dots) computed in *ABA6-2013* (abscissa, 414 regions) and *Kang-2011* (ordinate, 16 regions). It also shows the distribution of the baseline random distribution that was generated using permutation test (Fig 2A, dark dots, see Methods). We further tested that the effect was robust across subjects (see Methods and supplemental S7 Fig) and compared the results obtained with the BRO index to those obtained with ANOVA (see Methods).

In the *Kang-2011* dataset, Fig 2A and 2D, 66% of the genes have significant BRO scores (same as with the triplets method). The BRO scores for the two datasets are significantly correlated across the two datasets (Spearman **ρ** = 0.53, *p*-value < 10^-16^, n = 16947, Fig 2A). The differences between the two datasets in the fraction of genes that reach the significance threshold is due to the smaller number of tissue samples in *Kang-2011* and due to the limited coverage of non-cortical regions, since only 144 samples out of 491 (29%) were from non-cortical regions in *Kang-2011*, compared to 2187 out of 3702 (59%) in *ABA6-2013*. See full details in supplementary S1 Table. We provide BRO scores for all genes as a supplementary data file (S1 Data). We also quantified BRO scores in mouse, but due to multiple differences between the datasets, direct comparison of the results is hard (S1 Text).

Variability across regions and samples may be due to tissue-wide effects such as fluctuations in sampling, biases in cell density or variability in cell-type proportions, and these may be correlated with the developmental ontology. To test for a possible effect of cell density fluctuations across samples, we repeated the experiment after scaling each sample by its mean expression across genes. Fluctuations in the mean expression across samples were small (coefficient of variation, CV = std/mean = ~2.5%) and did not have a significant effect on BRO scores (Spearman **ρ** = 0.997 comparing with and without scaling). Similar results were obtained when normalizing to a set of highly-stable genes (see Methods) (CV = ~3%). This type of normalization is effective when expression of the gene is highly stable and the relation between cell density and number of transcript is largely linear.

As a second control, we aimed to test if BRO scores simply reflect the proportions of glia-neuron mixture, which varies across the brain. Since estimating cell-type proportions is challenging [25], and not yet available in human, we tested the relation between BRO-agreement scores and correlation with known cell-type specific markers, by quantifying how well the spatial variability of a gene can be explained by known neuronal and glial markers (see Methods). For neuronal markers, the median explained variance was 0.14 (median absolute deviation of 0.12, maximum explained variance = 0.26), and similar results were obtained with glial markers. One interpretation that is consistent with these finding, is that the mixture proportions of cell types in a sample, based on the markers available to us today, has a limited explaining power of spatial variability. It should be noted however, that cell type specific markers have both a limited sensitivity and limited specificity, and do not reflect perfectly cell type proportions. These results suggest that BRO-agreement is not a mere reflection of neuron-to-glia mixture proportions.

The transcriptome of the cerebellum is known to differ substantially from that of the rest of the brain [5,21,33]. This is apparent in Fig 1C, where the cerebellum is well separated from other regions. To test how strongly the cerebellum contributes to the high BRO agreement scores, we recomputed the scores while excluding cerebellar samples. Even with cerebellar samples excluded, 90% of genes are BRO-significant. An intuition for the robustness of the BRO can be gained from a principle component analysis (PCA) in a subsequent section. The non-cerebellar structures are aligned along an anterior-posterior dimension when projected onto the first two principal components. The order along this axis agrees with their place of origin in the neural tube and is captured by the BRO-score.

The BRO-agreement score reflects how strongly adult expression pattern of a gene may be driven by the ontology, which was coarsely based on brain development. It is natural to test how BRO-agreement scores change during life. We therefore computed the per-gene BRO-agreement scores in subjects of multiple ages based on the data of [6]. Samples were grouped as in [6], and covered postnatal and embryonic age groups starting at 13 post-conception week, all having a common set of brain regions. When considering ages from late embryonic development to adulthood, the mean BRO score follows an “hourglass” pattern, with BRO scores being lowest around birth (Fig 2E). This developmental pattern is in agreement with previous studies which analyzed aerial variability in the mouse [21] and human [19] brain. Based on these previous studies, the elevated BRO scores in embryonic development are due to higher areal variability in neural and brain development functions, while the postnatal rise in BRO scores is due to elevated variability in signaling and plasticity functions. The life-long BRO-agreement profile has two major differences from previous studies, namely, the BRO scores of both very early and very late stages are significantly lower, deviating significantly from the hourglass pattern. These differences are captured here because the scores are computed separately for very early embryonic and very late-life stages.

### Functional characterization of genes and their BRO-agreement scores

What genes and functions achieve the highest BRO scores? To answer this question, we first tested functional enrichment of gene ontology (GO) categories [34]. We used a threshold-independent approach based on ranking genes by their BRO (mHG [35]). The top enriched biological processes (S4 Table) are all brain related, and mainly belong to two families of functions: cell-to-cell signaling like synaptic transmission, and development-related categories like neuron differentiation and neurogenesis. The first family, genes in cell-signaling categories, included both genes from generic signal transduction pathways and receptors of more specific neuromodulator systems. For instance, the two genes with highest BRO score in synaptic transmission were *RASGRF2* –which coordinates activation of MAPK signaling, and *CAMK2A*—which is involved in calcium signaling). The 3^rd^ and 4^th^ genes in that category were genes coding for serotonin receptors *HTR2A* and *HTR4*. Finally, some of the top ranked genes are related to brain-related disorders: G-protein signaling regulator *RGS4*, serotonin receptor *HTR2A* and the postsynaptic protein encoder–*NRGN* are related to schizophrenia. *HTR2A*, *CHH9* are related to autistic disorders.

To understand better which genes achieve the highest BRO scores, we further studied gene families and functions that are of particular interest: genes expressed in specific cell types, and genes involved in regulation of brain development.

**Fig 3:**
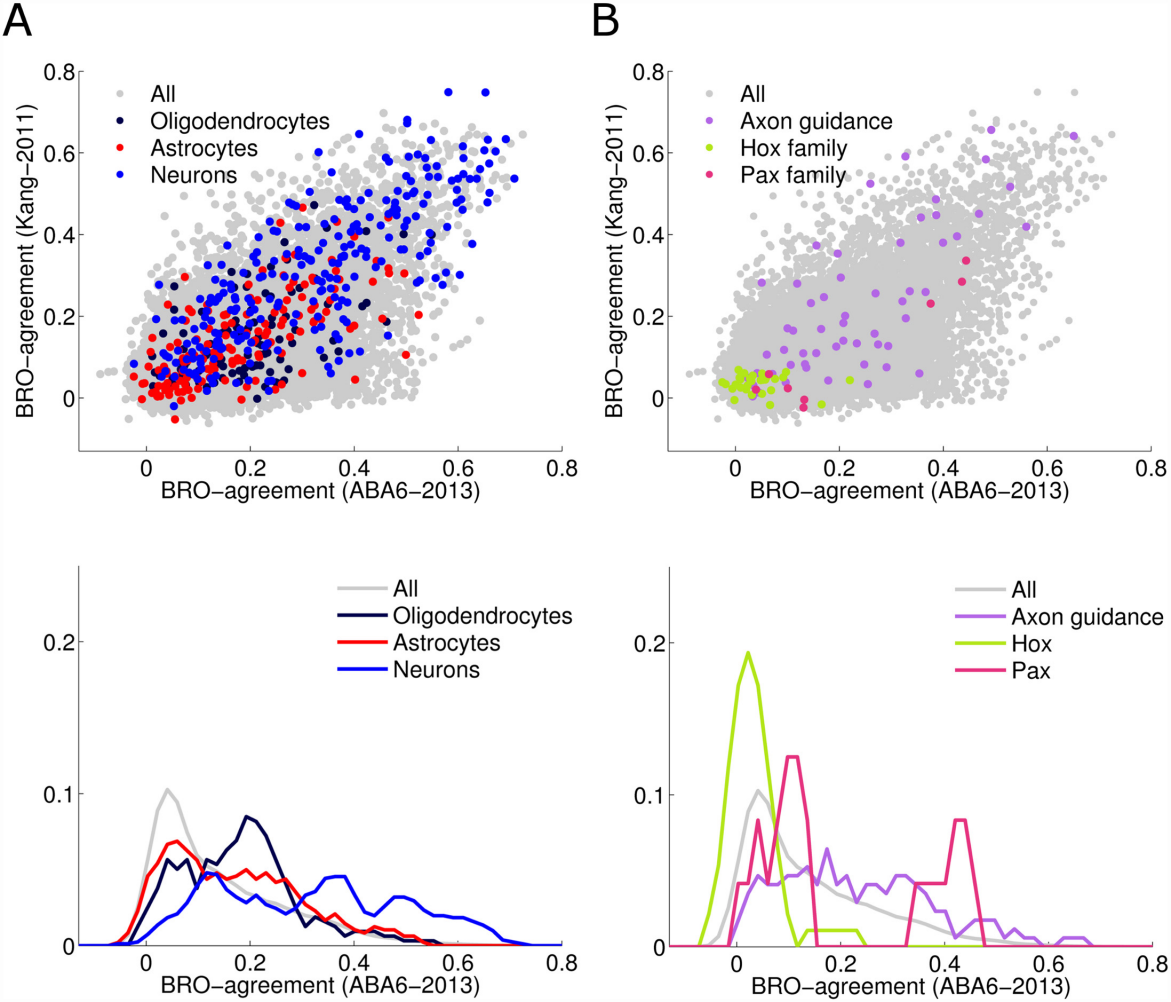
The distribution of BRO-agreement scores on different subsets of genes. The two top panels show a scatter plot of BRO scores in *ABA6-2013* and *Kang-2011*. The corresponding lower two panels show the (marginal) distributions in the *ABA6-2013* dataset. **(A)** Cell-type specific genes have higher agreement scores than all genes (Wilcoxonon tail test; neurons median = 0.33: *p*-value < 10^−70^ oligoodendrocytes median = 0.19: *p*-value = 10^−5^, astrocytes median = 0.16: *p*-value = 10^−3^). **(B)** Axon guidance genes receive higher scores than general genes (Wilcoxon median = 0.21; *p*-value = 10^−7^). Hox genes are less in agreement with region-ontology than the full set of genes. 21 Hox genes are BRO significant (67%) (compared to the randomized scores, with alpha = 0.01). *PAX2*, *PAX3* and *PAX6* obtain high BRO scores.

First, it has been suggested that genes that are expressed distinctively in specific cell types, contribute significantly to expression differences among brain regions [24]. Specifically, the combined expression of neuronal gene markers was shown to correspond to the major subdivisions of the brain [24]. Cahoy et al [36] identified genes that are enriched in specific cell types in the mouse brain and can be used as cell-type specific markers. Operating under the assumption that the human orthologs of those markers have preserved spatial expression patterns, their BRO-scores provide a way to test quantitatively how strongly neuron-specific genes agree with the brain region ontology. Fig 3A depicts three subsets of cell-specific gene markers, all of which are in particularly strong agreement with the tree structure: markers for neurons, astrocytes and oligodendrocytes. Cell-type specific genes have higher BRO scores than other genes on average (Fig 3A bottom). Interestingly, neuron-specific gene markers agree more strongly with the region-ontology than the average gene (*p*-value < 10^−70^,Wilcoxon comparing the median of the distributions of BRO scores of neuron-specific genes, median = 0.33 and the general population of genes, median = 0.11) while oligodendrocytes-specific and astrocyte-specific markers are far less so (oligodendrocytes median = 0.19, Wilcoxon, p-value = 10^−5^, astrocytes median = 0.16, Wilcoxon, p-value = 10^−3^). These results are in agreement with Ko *et al*. [24,37,38], which showed that the combined expression pattern of genes that are cell specific agrees with the region-ontology. The analysis above extends their results by showing that the region-ontology agreement occurs at the level of individual genes and is prevalent across cell-specific markers (neurons: 270/271 which are 99% of the neuronal markers are BRO significant; astrocytes: 151/160 which are 94% of the astrocytes markers; oligodendrocytes: 103/106 which are 97% of the oligodendrocytes markers).Using the same set of markers, Tan *et al*. showed that genetic markers for neurons and oligodendrocyte are on the opposite ends of the first principal component [37]. This means that while neurons and oligodendrocyte are similar in that they both agree with the developmental ontology, they also show a very distinct pattern of spatial expression.

Fig 3B depicts the distribution of BRO scores for three gene families involved in regional specificity during brain development: axon guidance genes, Hox genes, and Pax genes. Genes involved in early brain developmental have been shown to have regional expression patterns in the adult [39,40][23]. Here, we find that genes involved in axon guidance have very high BRO scores (Higher than the average gene, Wilcoxon, median = 0.21 *p*-value = 10^−7^) (Fig 3B bottom). This suggests that beyond their embryonic role, genes involved in axon guidance may assume other functional roles in the adult brain. Second, Hox genes play a major role in anterior-posterior patterning across the body and across the brain during development and largely retain these patterns in the adult body [16]. Their role in the adult brain is less clear. Here we find that many Hox genes have BRO scores above the random set, but on average, their scores are lower than the average gene (Wilcoxon, median = 0.03 *p*-value = 10^−9^). This suggests that unlike other gene groups discussed above, Hox genes are less involved in regional patterns in the adult brain. These view is also supported by Takahashi et al. which observed that while Hox genes show an expression gradient through the entire adult body, only one third of Hox genes are differentially expressed in brain-specific tissues [16].

Finally, we examined Pax genes. These genes are involved in early regionalization of the embryo brain and were suggested to play a role in differentiation and maintenance of specific subsets of cells in the adult brain [41,42]. It has been shown before that genes important for brain developmental have regional expression patterns in the adult [39,40], including genes involved in brain connectivity [23].

Here we find that three Pax genes, *PAX2*, *PAX3* and *PAX6*, obtain significantly large BRO scores (Fig 3B). Interestingly, *PAX6* is a major determinant of regionalization in the mammalian brain [9,10,42]. It was shown to be essential to cortex development, to mark cortex regionalization and to regulate radial migration of neuronal precursors [39,43,44]. The differential areal expression pattern of *PAX6* in the adult raises the hypothesis that *PAX6* continues to play a region-specific role in the adult brain.

### Evolutionary gene age and BRO-agreement

The above results suggest that spatial regionalization of human brain expression is present both in brain-specific functions and also in more generic processes that can be found in simpler organisms. Importantly, spatial regionalization of the nervous system is not unique to mammals or vertebrae, and some of the mechanisms controlling spatial patterning are shared across evolutionary-remote species [45]. For instance, Hox genes, whose expression exhibit anterior-posterior gradients in mammals, also hold spatial information in species that diverged from the human lineage early in evolution [46–48]. The natural question therefore arises: how is brain regionalization of a gene related to the evolutionary age of that gene? For instance, one may hypothesize that genes with high BRO agreement would be genes those that evolved recently, in organisms having a nervous system similar to the mammalian brain.

**Fig 4:**
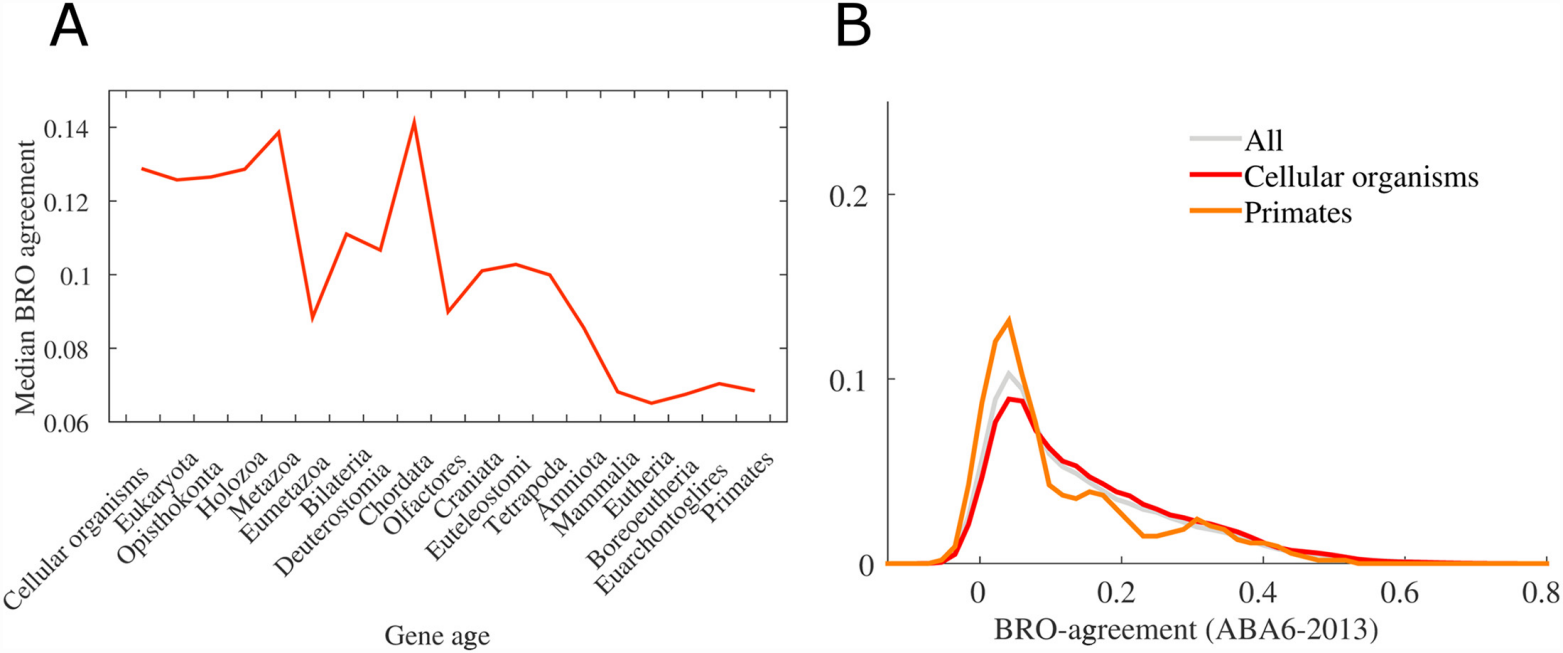
Evolutionary-older genes have on average higher BRO-scores. (A) The median BRO score as a function of the evolutionary age of genes. Older genes receive on average higher BRO scores than evolutionary recent genes. (B) Focus on the distribution of BRO scores for the oldest gene group and of the most recent (primates) gene group. Genes in the cellular organisim group have a median BRO-score of 0.129, while genes in the primate group have a median BRO-score of 0.068. The two distributions are signifatcly different.(Wilcoxonon two-tail test: p-value = 10^−6^).

To test this hypothesis, we compare the BRO index with an index quantifying the evolutionary age genes [49]. Surprisingly, we found that evolutionary-older genes have on average higher BRO scores than evolutionary-recent genes (Fig 4) (Cellular organisms: median = 0.129; Primates: median = 0.068, Wilcoxon *p*-value = 10^−6^). These older genes are also active in signaling pathways and other basic functions in the cell (S4 Table). The top BRO-scoring genes have orthologs across a wide variety of species, and participate in functions that are not specific to neural processes. Presumably, these genes were conserved as the result of a pressure to preserve these basic functions. For example, the gene *ENC1* encodes an actin-binding protein involved in regulation of neuronal process formation and in differentiation of neural crest cells. As another example, *CAMK2A* is involved in calcium signaling as part of the *NMDAR* signaling complex. At the same time, *CAMK2A* has an early evolutionary origin and has orthologs even in rice. On the other range of the evolutionary timeline, genes associated with speciation of primates obtain lower BRO scores on average. These results suggest that genes with strong BRO scores and spatial patterns are not necessarily specific to neural processes, but rather that the brain spatially tunes the expression of genes involved in fundamental molecular functions. With that said, it is also possible that these newer genes exhibit more refined differences across brain regions, but that these changes are not captured by the current coarse-scale analysis (also compare with [50]).

### Source of spatial variability in expression

Expression variability has many contributing factors, including subject-to-subject variability, regional variability and experimental noise. The above results suggest that the variability between brain regions is significant for most genes. But, how large is regional variability compared to other sources of expression variability? To answer this question, we used principal component analysis (PCA) to extract the main axes of variability in the data (see Methods). Interestingly, the PCA of the human expression was also analyzed previously by Tan et al. [31]. Tan et al. used PCA to embed genes in a low dimension space that preserved much of the *gene-to-gene* variability. In that space, they found that neurons and oligodendrocytes are on the opposite end of the first principal component. Here we address the complementary analysis, looking for the dimensions that preserve the *sample-to-sample* variability.

**Fig 5:**
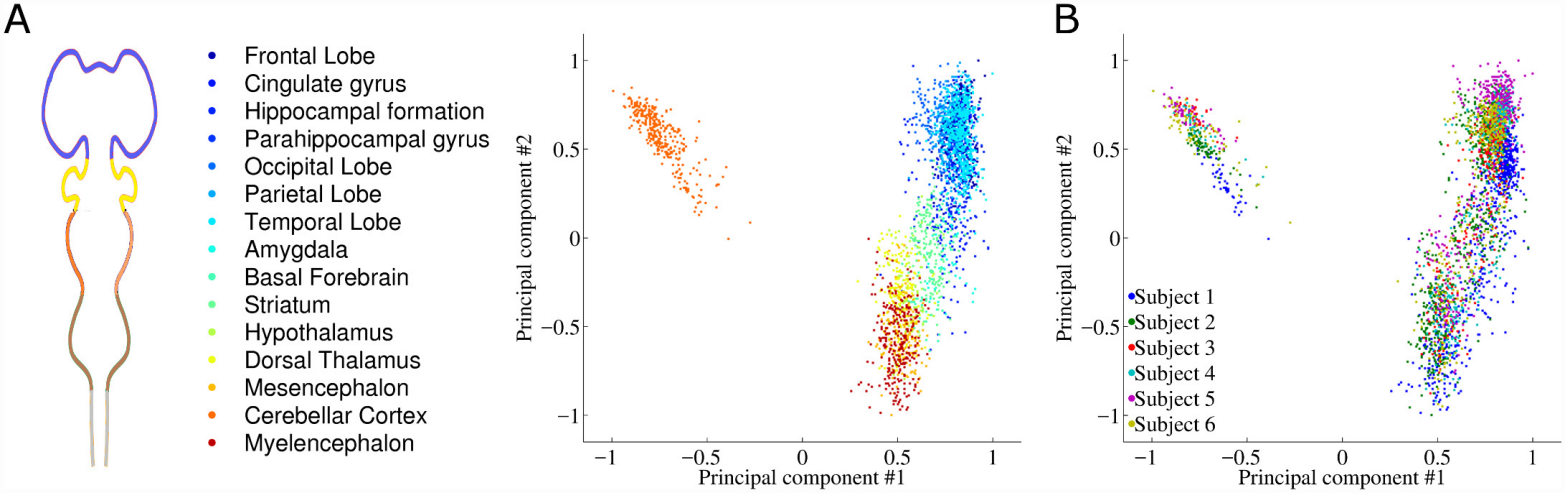
Projection of the samples from the human6 dataset on the 1st and 2nd principal componenets with two coloring schemes. (A) The samples are colored according to the position of the corresponding embryonic region, using the same color scheme as in Fig 1A. (B) The samples are colored according to one of the six donors.

Fig 5A shows the projection of brain samples onto the 1^st^ and 2^nd^ principal components, which together account for 34% of the variance (S3 Fig). Samples are colored by the brain region from which they were taken. Brain regions are well separated in this projection, in a way that matches the anterior-posterior axis and the BRO. The isolated cluster of samples on the left belongs to the cerebellum, which is well known to exhibit a unique molecular and cellular organization [21,51,52]. This analysis shows that the BRO is a major determinant of variability in human brain transcriptome.

As a comparison, Fig 5B shows the same projection on the two top PCs, but this time the samples are colored by the subject from which each sample was taken. Expression differences between people are pronounced mostly in frontal regions (top right samples), but are dramatically weaker than the differences between brain regions. Subject-to-subject differences are more pronounced when projecting on the 3^rd^ and 4^th^ principal components (S2 Fig).

To quantify the relative contribution of subject identity and region of origins to expression variability, we computed the fraction of variance explained by these two features. For every gene, we examined it expression across samples separately and computed the fraction of explained-variance (See Methods) (S8A Fig). The subject-identity explains 0.13 (+/- 0.12) of a gene’s expression-variance, while the region explains 0.28 (+/- 0.21) of a gene’s expression-variance. Together, both sources explain nearly half of the sample variability (median at 0.43 +/- 0.18). Region and subject identity explain “different” component of the variance: the fraction of variance explained by region is inversely correlated with the variance explained by subject id. (Spearman *ρ* = -0.51 S8B Fig). Using the same dataset, Hawrylycz et al. recently took the complementary track and searched for stable expression-patterns across subjects [53]. They showed that genes with conserved patterning across subjects display strong relationships to anatomical structure, functional connectivity and other features of the human brain.

### BRO agreement and spatial variability in paralogs and functionally-related genes

With many genes exhibiting spatial expression that matches the developmental origin of brain regions, the question remains if and how expression variability is used by the brain to tune the functional properties of cells and circuits. One particularly interesting aspect of such tuning is how the brain controls the expression of similar genes, including paralog and other functionally-related gene pairs. In many cases, the brain is known to switch from expressing one paralog variant to another variant. Such switches have been studied mostly in the context of development and synaptic pathways, including the widely studied switch in NMDA receptors from subunit *NR2A* to *NR2B* [54–57]. These developmental switches can be traced to occur within a brain region, and in some cases well after birth [54,56]. Here we study *spatial switching* in pairs of genes, where genes coding for different protein variants are expressed in different brain regions.

We set to study the relation between spatial expression switching and developmental origin of regions, using the per-gene BRO-agreement score. To study fine spatial tuning, we aimed to focus on pairs of genes that share similar functions. To collect such gene pairs we used two approaches. First, we used a set of paralog genes defined by Ensembl (denoted *ensemble-based paralogs*). Second, to further focus on genes with putative similar function, we collected pairs of genes that share the same functional role in cellular pathways, as captured by KEGG. We also required that these gene pairs have a significant sequence similarity and denote this set *Kegg-based pairs* (see Methods). Both sets were restricted to brain-related synaptic pathways.

**Fig 6:**
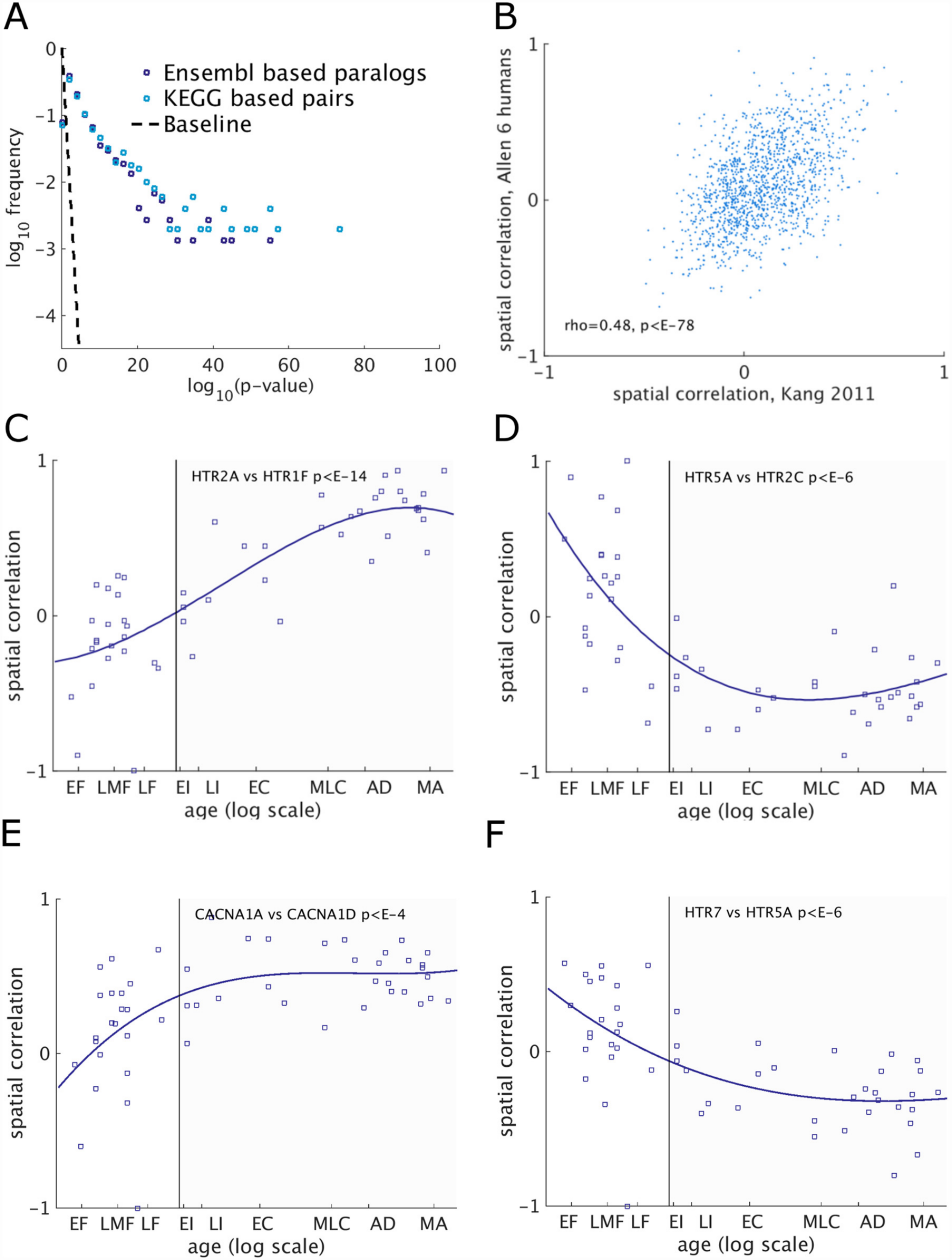
**(A)** Distribution of gene pairs with anti-correlated spatial expression. **Kegg-based** gene pairs include 1496 pairs with (1) sequence similarity > 30%, and (2) sharing a sub-component in one of 17 KEGG synaptic pathways (see Methods). **Ensembl-based paralogs** include 3503 pairs of paralogs (as defined by Ensembl) where both genes in a pair are included in one of same 17 pathways (see Methods). Baseline corresponds to the distribution expected at random. **(B)** Consistency of spatial correlations across two datasets *ABA2013* and *Kang-2011*. The spatial correlations of paralog pairs across the two dataset, show a significant agreement (Spearman ρ = 0.48, *p*-value < 10^−78^). Each point correspond to the median correlation across adult subjects, in one gene pair (total of 1496 pairs). **(C-F)** Examples of development of spatial correlations in the Serotonin system. **(C)** The pair of genes coding for Serotonin receptors *HTR2A* and *HTR1F* exhibit a continuous rise in spatial correlation, riding from slightly negative in early embryonic development to strong positive correlation. **(D)** The pair of genes coding for Serotonin receptors *HTR5A* and *HTR2C* show a sharp transition from positive to negative spatial correlation in early development, which is then preserved through life. **(E)** The paralogs *CACNA1A* and *CACNA1D* exhibit a rise in spatial correaltion **(F)** The paralogs *HTR7* and *HTR5A* show a continuous change in spatial correation, from positive corealation during embryonic development to negative one at adulthood.

We first compared the distribution of spatial correlation strengths of similar gene pairs, quantified by log(p-values) on *ABA6-2013* data. We found that both the KEGG-based gene pairs and the Ensembl-based paralogs are significantly more spatially anticorrelated than random gene pairs (Fig 6A). Furthermore, KEGG-based gene pairs are more anticorrelated than Ensembl paralogs in these pathways (Fig 6A). The spatial correlations of KEGG-based pairs are fairly consistent when compared to the correlation using the same gene pairs in adult brains from the *Kang-2011* dataset, considering they were measured by different labs and in different brain regions (Fig 6B).

Next, we compared the strength of spatial correlation of each pair of genes with their combined BRO-agreement scores, and found a strongly significant correlation between the two (KEGG-based set, Spearman with log(*p-*value), ρ = 0.36 *p*-value < 10^-42^, n = 1496). More surprisingly, when considering anti-correlated gene pairs, pairs with high BRO-scores tend to be more strongly anti-correlated (Spearman rho = -0.27 *p*-value<10^−9^ n = 1496). This effect is not simply due to some pairs having more variable expression across the brain, since the dependency on BRO is significantly stronger (*p*-value <10^−42^) than the dependency on spatial variability (*p*-value < 0.01, quantified using the standard deviation across samples). One interpretation of these findings is that the brain tunes the expression of pairs of functionally-related pairs of genes, such that they are expressed differently in brain regions, and that this tuning is in strong agreement with the developmental origin.

Together with the BRO results, these findings suggest that correlated spatial expression may be formed early in development. To test this hypothesis we computed the spatial correlations for each subject in the *Kang-2011* data, which allows tracing how spatial correlations develop with age. We then searched for KEGG-based pairs whose spatial correlations follow a trends, and found that 17% of the pairs exhibit a significant trend (257/1496, FDR corrected *p*-value<0.01, F-test from fitting a linear regression model, as compared with the constant model). Far fewer pairs of Ensembl paralogs exhibit a significant trend (6.8% of the pairs 240/3503).

Fig 6C shows the top pair in the KEGG-based pairs set (*p*-value of trend < 10^−4^). It corresponds to a pair of Serotonin receptor genes, *HTR2A* and *HTR1F* from two different receptors (5-HT1, 5-HT2). Interestingly, their spatial correlation is negative prenatally and is around zero around birth. However, it continuously grows throughout life, reaching high positive correlation at adulthood. This pattern is interesting for several reasons. First, the gradual increase in correlations throughout life is not likely to be caused by changes in cell proportions, since there is a significant change in correlation between childhood and adulthood. Second, other genes in the Serotonin system exhibit different patterns. Fig 6D shows a pair of genes coding for Serotonin receptors *HTR5A* and *HTR2C*. Here the early embryonic positive correlation is replaced by a negative correlation around birth, which remains quite stable during life. Fig 6E and 6F show similar patterns in two pairs of Ensembl paralogs, *CACNA1A* vs *CACNA1D* two genes coding for Calcium channels, and *HTR7* vs. *HTR5A*, two serotonin receptors. *HTR7* is known to be involved in both early and post-natal development [58].

One possible interpretation of the prevalence of high BRO-agreement scores is that the expression patterns of many are determined early in development, and are preserved through life and in the adult brain. Alternatively, it is also possible that gene-expression changes in a dynamic way through life, but keep following patterns that agree with the embryonic origin of regions. To test these two hypotheses, we quantified the relation between the strength of expression changes of pairs through life, and the BRO scores of the gene pair. We find that the two are positively correlated (ρ = 0.14, p-value<10–7, S9A Fig, for KEGG-based pairs, and, ρ = 0.17, p-value<10–21, S9B Fig for Ensembl-based paralogs), namely, pairs of genes with higher BRO scores actually tend to exhibit more changes in their spatial correlations, consistent with the second hypothesis. These results are consistent with the view that spatial expression patterns in the adult are not a mere reflection of the brain structure as determined in early development, but are tuned to use genes coding for different protein variants in a differential way across the brain.

## Discussion

To characterize the areal patterns of gene expression in the human brain, we analyzed two datasets of mRNA expression from post-mortem adult donors. For each gene, we computed an index that measures how its expression pattern agrees with a hierarchical ontology of brain-regions, based on their developmental origin. We find that 92% of human genes exhibit an expression pattern that significantly agrees with the known brain-region ontology. The fact that such a large fraction of the human genome is differentially expressed across brain regions suggests that control of expression in the brain is largely region-specific.

When focusing on genes that are expressed specifically in neurons, glia and oligodendrocytes, we find that cell-type specific genes tend to strongly agree with the tree-structured ontology. This suggests that not only do these markers differ between regions, as suggested by Ko *et al*. [24], but that they also follow a specific pattern of expression which corresponds to the embryonic origin of the region and to a larger extent than the average gene. Interestingly, significant BRO scores are not limited to neurons, which are often known to differ across brain regions, but are also observed in glia-specific genes, which are often viewed as performing brain-wide and generic functions.

Having adult expression patterns that strongly agree with the developmental brain region ontology could have various interpretations. First, adult spatial expression patterns could be determined by the embryonic origin of each region, for example because brain regions differ by their cell-type profiles, or due to the expression of region-specific markers. Alternatively, adult expression may reflect delicate tuning of expression where different brain regions utilize different protein variants, optimized for the function of each brain region. We find evidence that support the second alternative. First, gene with high BRO-scores tend to change their expression more during development. Second, pairs of functionally-related genes (participating in a similar role in synaptic pathways) have stronger spatial anti-correlation than paralogs in those pathways. Finally, in those pairs of functionally related genes, pairs with higher BRO scores tend to have stronger spatial anti-correlation.

The approach we presented has various limitations. Transcriptome data measured from brain tissues involves a mixture of various cell types whose proportions and conditions are not known. Developing demixing approach to infer proportions from the mixture [25] is an important challenge, can be based on single-cell transcriptomics (as in Darmanis et al 2015), and is likely to significantly change our understanding of brain transcriptome.

Genes involved in patterning and axon guidance clearly exhibit regional patterns during early development [59]. The above results show that their expression continues to be governed by the region ontology in the adult brain, long after their developmental role has been completed. As one specific example, consider a gene from the top BRO-scorers in the *ABA6-2013* dataset, *FEZF2* (forebrain embryonic zinc finger protein 2), a transcription repressor involved in specification of subcerebral projection neurons [60,61]. *FEZF2* is believed to play a role in layer and neuronal patterning of subcortical projections and axonal fasciculation and was shown to regulate axon targeting of layer 5 subcortical projection neurons, where axons of *FEZF2* deficient mice failed to reach their targets [62]. In the adult human brain, our results show that *FEZF2* retains strong areal differences in adulthood, and is strongly expressed in the cortex, less so in the midbrain and the least in the hindbrain (S6 Fig). Indeed, the mouse variant of FEZF2 is known to be expressed in adult projection neurons [62]. Importantly, these results suggest that *FEZF*2 assumes another functional role in the adult cortex. Indeed, it has been shown that projecting neurons in the mouse motor cortex expressing *Fezf2* have distinct physiological characteristics [63]. The abundance of genes that retain their areal differential expression in adulthood suggests that this may be the general case, and many genes that play a role in brain development later assume new roles that affect the function of the adult brain.

The fraction of genes having distinct areal expression pattern has been previously estimated using a different method (ANOVA). In the *Kang-2011* data, it was found to be on the order of hundreds of genes in the adult human brain (Pletikos *et al*. [19], Fig 2B). In the *ABA6-2013* data 84% of the genes were found to have differential expression across brain regions [5]. ANOVA estimates are sensitive to differences in the mean expression of regions, regardless of the region ontology, and could capture genes whose expression pattern in some brain regions is different from others. As such, they are more sensitive to genes that are uniquely expressed in one or few region. The BRO-score can therefore be viewed as a complementing measure, which is sensitive to global areal-differential expression that is consistent with the brain region ontology.

The fact that the expression of most genes in the adult brain is governed by earlier development stages suggest that many studies which deal with regional differential expression should be carefully interpreted. For example, combining samples taken from ontology-distant regions would lead to large expression variance, reflecting the developmental origin of the structures tested. Furthermore, areal differential expression should be measured compared to a baseline expression profile that takes in to account the region ontology.

The results in this paper suggest that spatial expression patterns in the adult human brain are controlled in a way that follows the embryonic origin of regions, but at the same time that spatial patterns of related genes may change during development in a correlated way. It remains to be discovered which transcription control mechanisms maintain these distinct areal expression patterns.

## Methods

### Gene expression measurements

We analyzed gene expression data from two sources. First, a set of 3702 microarrays provided by the Allen Human Brain Dataset (*ABA6-2013*) from *human*.*brain-map*.*org* [5]. We mapped 58692 microarray probes to genes based on mapping provided by the Allen Institute. When multiple probes were available for a gene, we selected the probe that was most consistent across the 6 human subjects; suggested by *human*.*brain-map*.*org*. Specifically, when we analyzed genes with multiple probes, we first computed the expression correlation across regions of each probe, then averaged the correlation scores across all pairs of subjects and chose the probe that was most correlative. Overall, we analyzed 20773 transcripts. The number of samples per donor ranged from 363 to 946, for a total of 3702 tissue samples.

The second dataset was a set of 1340 microarray samples collected by Kang and colleagues from 57 postmortem brains containing expression values for 17565 genes [6]. We refer to this dataset as *Kang-2011*. We limited the analysis to donors that are older than 12, yielding a total of 20 donors and 491 tissue samples.

The Kang dataset was also used for the analysis of BRO-agreement over development (Fig 2E). For this analysis we also used the pre-natal subjects and early-childhood subjects. The subject ages range from 13 post conception weeks to 82 years. We grouped the subjects into 12 age-groups following the original Kang paper, and computed BRO scores per age group.

### Brain region ontology

We used the brain region ontology hierarchy provided by the Allen institute *human*.*brain-map*.*org* containing 1534 regions organized in a hierarchical manner. From the full set of regions we used two ontologies: A fine region-ontology with 414 regions which had measurements that were associated with them, and a coarse region-ontology with 16 brain regions. The list of 16 gross regions is given in supplemental S2 Table. The coarse part of the ontology (upper part in Fig 1A) was largely based on brain development while the fine parcellation of regions was based more on cytoarchitecture. The results we report are based on the coarse 16 region-ontology. We report below results for both coarse and fine grained ontologies. Measurements from the Kang-2011 dataset were obtained from 16 regions. We mapped those regions to 16 regions of the Allen ontology, and the mapping is given in supplemental S3 Table.

### BRO-agreement scores

The BRO-agreement score was computed separately for each gene as follows. For each pair of samples *(a*,*b)*, we define their tree similarity as the distance (number of edges) between the regions in the ontology hierarchy tree *d*_*tree*_*(a*,*b)*. We define their expression similarity as the absolute difference between the expression values of the two samples for the current gene (i)—*d*_*expression*_*(a*_*i*_,*b*_*i*_*)*. We computed the two distances over all pairs of tissue samples, and computed the Spearman correlation between the two as the BRO score.

$$BRO\_score(gene\;i)≔spearman\;corr_{(a,b)\in all\;sample\;pairs}(\;d_{tree}(a,b),\;{\;d}_{expression}(a_{i},b_{i})\;)$$

To generate random scores, we calculate Bro-agreement scores of permutated vectors. Genes with a BRO-score above the top 1% of the permuted scores were considered significant.

We also tested a second ontology-agreement score based on triplet ranking. We randomly selected 10^6^ sample triplets (a, b, c) and computed the fraction of times that a triplet is ranked with the same ordering in both the tree and the expression distance measures:

$$BRO\_triplet(gene\;i)≔\frac{\#(d_{tree}(a,b)<d_{tree}(a,c))\;\&(d_{expression}(a,b)<d_{expression}(a,c)))}{\#(d_{tree}(a,b)<d_{tree}(a,c))}$$

This score gave similar results which are not shown here.

To handle biases that could arise from different scales in the samples we also checked a normalized version of the Bro-agreement. In this normalized version we first normalized each sample to zero mean and unit variance and then computed the BRO-agreement. The results were robust to this change and we choose to present the un-normalized BRO-agreement scores. To handle biases that could arise from the number of regions in the ontology, we used two different granularities of the ontology tree. The first uses 16 gross regions and the second uses the entire tree (414 regions).

### Combining scores from multiple subjects

We tested two ways to combine expression measures from multiple subjects into a single BRO score. First, we simply aggregated all samples of all subjects from a given region, and computed the BRO agreement score. Second, in the *ABA6-2013* dataset, the number of samples per subject is large enough, such that a BRO score can be computed separately for each subject. We then computed a global BRO score of a gene as the average over the 6 individual-subject BRO scores. 94% of the genes were significant compared to random, according to the first score, and 92% according to the second method. For consistency with the *Kang-2011* data, the figures use the first method. We report the more conservative estimate of significant genes as 92%.

We computed BRO-scores which uses the fine region-ontology. The upper branches in this ontology are more developmental oriented and the lower branches are more cytoarchitecture-driven. Using this ontology 89% of the genes are BRO significant. The BRO scores of the fine region-ontology and of the coarse 16 region-ontology are very similar with a spearman correlation of 0.98.

To test if these results are sensitive to the number of region available in ontology tree, we repeated the analysis of the *ABA6-2013* dataset at a coarser resolution of 16 regions. Using this coarse resolution, 95% of the genes were BRO significant. To test if the number of BRO significant genes is sensitive to the number of available samples, we randomly subsampled subsets of size 500 samples, which decreased the fraction of BRO significant genes to 51% ± 2% (supplementary S1 Table).

### Robustness across subjects

The percent of BRO-significant was tested for robustness using the ABA6-2013 dataset, where hundreds of samples are available for each subject. For each gene, we calculated its BRO-score but this time for each subject separately. The fraction of genes with a significant BRO score (p-value < 0.01) is stable across the individual subjects, yielding 89%, 90%, 76%, 91%, 83% and 86% (supplementary S7 Fig). The fraction of significant genes at the group level is slightly higher; suggesting that the groups score manages to remove some of the inter-subject noise.

### ANOVA analysis

Expression variability of a single gene across regions is sometimes captured by comparing the mean expression level in each region using ANOVA [5,6]. This approach would find a gene as significant even if it is differentially expressed in a single region, or if it is expressed in a set of regions regardless of their position in the ontology tree. Hence in principle, the BRO agreement index poses a stronger requirement of agreement with the ontology. When computing ANOVA across 414 regions of the *ABA6-2013* dataset, and using the average scores for each of the 6 subjects, 86% of the genes (17895 out of 20773) were significantly differentially expressed across regions (FDR-corrected *q*-value < 0.01), 83% of these genes (17266 out of 20773) were also BRO-significant. 8% of the genes (1719 out of 20773) had BRO-significant scores but not ANOVA-significant, most likely because the BRO index combined weak affects across multiple nodes of the tree.

For each gene, we computed one-way ANOVA on samples expression levels. The *p*-values reported are under the null hypothesis that samples are drawn from regions which have the same mean expression. We computed ANOVA separately for each human subject and report the average ANOVA score across subjects for each gene. Similar to the BRO scores we performed ANOVA on the fine grained ontology and on the coarse ontology. We then corrected the *p*-values for multiple comparisons using FDR.

### Cell type-specific markers

For the genome wide analysis of BRO scores We used the human orthologs of the set of genes characterized by Cahoy et al. [36], who used microarrays to profile expression patterns in purified populations of neurons, astrocytes and oligodendrocytes. For testing how spatial variability could be explained by cell-type specific markers, we used a set of known markers collected from various sources including [36]. For neuronal markers we used *EMX1*, *MAP2*, *GRIA2*, *DLG4*, *DLG3*, *NRGN*, *STMN2*, *SYT1*, *CELF4*, *CELF5*, *CELF6*. For glia markers we used *GFAP*, *MBP*, *SLC1A2*, *SLC1A3*, *DLG4*, *DLG3*, *MAP2*. To compute the dependence of spatial variability on those markers we fitted a quadratic function for each of the genes separately using a least square loss, and computed the explained variance R^2^, compared to a constant model.

### Sets of gene pairs

We studied two sets of gene pairs: ***Ensembl paralogs*** and ***KEGG-based*** gene pairs. For the first set, we used the paralogs available from Ensembl (*ensembl*.*org*, May 2016), and limited to gene pairs that had an Entrez id and were included in synaptic and brain related pathways as described by KEGG. Specifically, these included 17 pathways with KEGG accession numbers 04020, 04724, 04725, 04726, 04727, 04728, 04730, 05010, 05012, 05014, 05016, 05030, 05031, 05032, 05033, 05034, 04080.

Second, for ***KEGG-based*** gene pairs, we created a set of gene pairs designed to capture functionally-related genes, by collecting gene pairs that reside within the same functional element in KEGG pathway repository. These KEGG elements group together proteins with common functionally and interaction partners. We found these KEGG elements to be usually more functionally-coherent than protein families, and at the same time less specific than protein sub-families. We further required that pairs have sequence similarity above 30% (See also [64]).

### Trends in spatial correlation

To find pairs whose spatial correlation has a trend, we fitted a linear regression model with least square loss with age as the predicting variable and spatial correlation as the predicted variable. Significance of the trend was measured based on the F statistic of the explained variance and was FDR corrected for multiple hypotheses.

### Hox, Pax and axon guidance genes

We used the set of genes that belong to the human axon guidance pathway. The set was manually curated by KEGG (*www*.*genome*.*jp/kegg*). The Hox genes used are: *HOXA1*, *HOXA10*, *HOXA11*, *HOXA2*, *HOXA3*, *HOXA4*, *HOXA5*, *HOXA6*, *HOXA7*, *HOXA9*, *HOXB1*, *HOXB13*, *HOXB3*, *HOXB4*, *HOXB5*, *HOXB6*, *HOXB9*, *HOXC10*, *HOXC12*, *HOXC13*, *HOXC4*, *HOXC5*, *HOXC8*, *HOXC9*, *HOXD1*, *HOXD12*, *HOXD13*, *HOXD3*, *HOXD4*, *HOXD8*, *HOXD8*, and *HOXD9*. The Pax genes used are: *PAX1*, *PAX2*, *PAX3*, *PAX4*, *PAX5*, *PAX6*, *PAX7* and *PAX8*.

### Highly stable gene

A set of 11 genes collected by Eisenberg et al. [65] and available at (http://www.tau.ac.il/~elieis/HKG/).

### Hierarchical clustering analysis

We used agglomerative hierarchical with average linkage and Euclidean distance over 3702 samples from *ABA6-2013* obtained from six subjects. Samples from the same brain regions were first averaged to create a single profile for each region.

### Principal component analysis

We used all 3702 samples from *ABA6-2013* to compute the covariance matrix of gene expression levels, and then computed the top principal component of the expression covariance matrix.

### Explained sample-variance analysis

For each gene, we evaluated how much of its expression variance (over samples) can be explained using two sources of information: The region the sample was taken from and the identity of the subject the sample was extracted from. The explained variance was computed for each gene by fitting linear model using each of these sources of information, and using both of them together.

### Gene enrichment analysis

For robustness, we combined two BRO-score of each gene by multiplying the BRO-score computed with the *Kang-2011* data with that computed with the *ABA-2013* data. We performed the ranked based enrichment analysis using Gorilla (http://cbl-gorilla.cs.technion.ac.il/).

### Gene age-index

*We used the gene age-index published by Domazet-Lošo and Tautz* [66].

## Supporting Information

S1 Table Sensitivity of the number of BRO significant genes in the ABA6-2013. Sensitivity to the number of regions in the ontology tree depth was assessed by using two ontologies with different resolutions: A fine-resolution ontology that contained 414 regions and a coarse-resolution ontology of 16 gross regions. Sensitivity to the number of available samples was assessed by computing the BRO score using random subsets of 500 samples.
(XLS)

S2 Table Names of brain regions and their corresponding symbols ABA ontology [31]. (XLS)

S3 Table Region notations. Mapping the notation of regions in Kang-2011 and in the Allen Institute region ontology ABA6-2013 [5,6].
(XLS)

S4 Table Top 10 ranking of GO biological processes enriched in genes with high BRO scores. Enrichment was computed using mGH [35] testing for enriched biological processes using the full ranked list of BRO-score genes. All top processes are brain-related, with high enrichment for cell signaling and neural development.
(XLS)

S1 Fig Correlation between tissues. Pearson correlation between expression vectors of all pairs of tissue samples. Samples are ordered first by gross region than by donor. Samples from regions that are close in the developmental region ontology are highly correlated in their expression profile. Within each region, samples are also correlated with other samples from the same donor.
(TIF)

S2 Fig Projection of samples from ABA6-2013 dataset onto the 3^rd^ and 4^th^ principal componenets as in Fig 5, with two coloring schemes. **(A)** Each point is a tissue sample. Samples are colored based on the position of the corresponding embryonic region. **(B)** Colors correpsond to donor identity. A significant fraction of the sample variance across the 3^rd^ and 4^th^ principal components is explained by subject-to-subject variablity.
(TIF)

S3 Fig Fraction of variance explained by the top principal components, as in S2 Fig. The first two principal components caputre 34% of the variance. Adding the 3^rd^ and 4^th^ principal components explain more than 51% of the sample-to-sample variance.
(TIF)

S4 Fig Distribution of cortex-BRO-agreement scores in *ABA6-2013* and *Kang-2011*. Color scheme and x-axis scale matche those of Fig 2. **(A)** A scatter plot showing BRO-agreement scores for the two datasets. Each light-grey dots corresponds to a single genes (a total of 17K genes). Dark-grey dots correspond to permuted data (see Methods). **(B)** Marginal distribution of BRO scores in the *ABA6-2013* dataset. In the *ABA6-2013* dataset, 11% of the genes (2207 out of 20773) are BRO-significant in the cortex. BRO score was also computed separately for each subject, using the *ABA6-2013* dataset. With these per-subject scores, the number of BRO-significant genes varied considerably across the six subjects (30%, 33%, 5%, 37%, 16% and 18%), and the correlation of BRO scores between subjects is on average lower (mean Spearman Correlation of cortex BRO scores of a pair of subjects is 0.23 ± 0.13, compared with 0.76 ± 0.08 for the whole brain). **(C)** Marginal distribution of BRO scores in the *Kang-2011* dataset. The large fraction of BRO-significant genes observed in *ABA6-2013* was not found in the *Kang-2011* dataset, where the two distributions largely overlap.
(TIF)

S5 Fig The distribution of cortical BRO-agreement scores on various subsets of genes. Color scheme and x-axis scale match those of Fig 3. **(A, B)** Neurons and astrocytes receive significantly higher agreement scores than all genes (Wilcoxon one tail test; neurons: *p*-value = 10^-9^, astrocytes: *p*-value = 10^−22^). Oligoodendrocyte genes are in less agreement with region-ontology than the full set of genes. Comparing these BRO scores to a randomized scores we find that 45% of the neuronal markers are cortex-BRO significant, 69% of the astrocytes markers are cortex-BRO significant and that 7% of the oligodendrocytes markers are cortex-BRO significant;). **(C, D)** Axon-guidance genes receive higher scores than genes on average (Wilcoxon one tail test, *p*-value = 10^−3^). Hox genes are less in agreement with region-ontology than the full set of genes (still significantly larger than random). *PAX2* and *PAX6* obtain high BRO scores.
(TIF)

S6 Fig FEZF2 (ZNF312) expression pattern corresponds with the brain region ontology. FEZF2 shows a clear transtion of its expression levels. The samples from the cortex show high experssion values where the samples midbrain has less and the samples of the hindbrain has the least expression. **(A)** The mean expression levels of the FEZF2 within different region in the human brain (the color scheme as consitant with that of Fig 1A. **(B)** We embed the samples in a 3D space using its MRI standartize location where the color of each sample shows the expression level of FEZF2. The scatter shows a transiation from high expression levels in the cortex to lower expression levels in the inner brain structures and to the hindbrain.
(TIF)

S7 Fig The distribution of BRO-scores computed separately for each subject in ABA6-2013. The percent of BRO-significant genes (*p*-value < 0.01) is stable when computed for each subject separately: 89%, 90%, 76%, 91%, 83% and 86%).
(TIF)

S8 Fig Region information explains more of the sample variance than subject identity. The joint information of subject id and region id explains almost half of the sample variance. For each gene, we represented the identity information as 1-hot-vectors, and computed the explained sample-variance by fitting a linear model **(A)** Distributions of explained variance across genes, as explained by region, subject or both. **(B)** The joint distribution of both the explained variance from region and from subject identity.
(TIF)

S9 Fig BRO score is positively correlated with pairs of genes which show a significant trend in development. Trend significance of change in spatial correlation through life was quantified using the standard F-test comparing residual of a linear regression model with a constant model. Pair BRO score is the minimum BRO scores of the two genes in the pair. **(A)** We computed the developmental trend for each pair of genes from the KEGG-based set. We found that BRO-agreement scores are (weakly but significantly) positively correlated with having a significant trend. Each dot corresponds to one brain related sequence-similar pair. 17% of pairs (257/1496) had FDR-corrected significant (q<0.01) linear trend of their spatial correlation, as illustrated in Fig 6. **(B)** Similarly, we found that the developmental trend of paralog pairs which are brain related is also positively correlated with the BRO score. 6.8% of the brain related paralog-pairs show a significant trend (240/3503).
(TIF)

S10 Fig **(A)** 2D scatter of BRO scores for genes with a matching homolog in mouse (Zapala et al.[22]) and human (ABA2013) and a random baseline **(B)** 2D heat map of the joint distribution of BRO-scores for mouse and human. **(C)** The distribution of BRO-scores in mouse and a random baseline, showing that the fraction of BRO significant genes is smaller in the mouse dataset (30%) than in humans.
(TIF)

S11 Fig **(A)** The Allen ontology used for the human analysis. **(B)** The Allen ontology used for the mouse analysis. Both follow ontologies first devide the brain into the 5 embrionic vesiceles and then go into more detailed regionalization. The leaf regions are not the same since the data was gathered in different experiments each with a unique focus.
(TIF)

S1 Text Supplemental analysis text. BRO in the human cortex and a comparsion between the BRO in human and in mouse.
(DOCX)

S1 Data A list of per gene BRO score. The table containt: Gene symbo, Gene Entrez-ID, BRO score that is the product of the BRO of ABA6-2013 and Kang-2011, BRO score computed from the ABA6-2013 dataset, BRO score computed from the Kang-2011 dataset. A mark per gene which indicates it involvment in one of these 11 classes: cell-cell signaling, synaptic transmission, neuron differentiation, neuron projection development, generation of neurons, axon development, neuron development, schizophrenia, autistic disorder, seizures,epilepsy, substance related disorders.
(CSV)
