# Supplementary figures and images for: On Expression Patterns and Developmental Origin of Human Brain Regions
Source: PLoS Comput Biol. 2016 Aug 26;12(8):e1005064. doi: 10.1371/journal.pcbi.1005064 (PMC5001727; doi:10.1371/journal.pcbi.1005064)

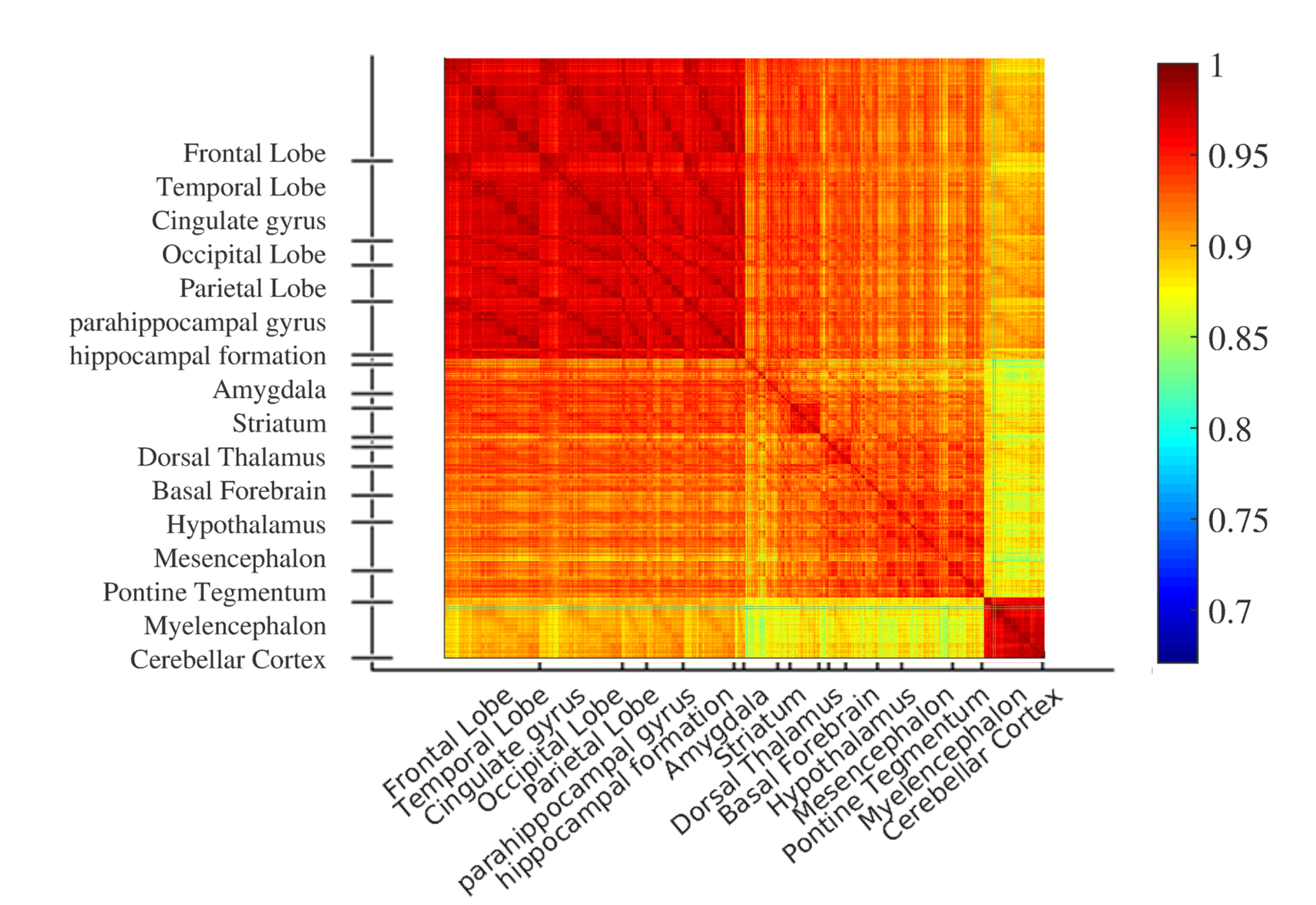

Supplement: S1 Fig — Pearson correlation between expression vectors of all pairs of tissue samples. Samples are ordered first by gross region than by donor. Samples from regions that are close in the developmental region ontology are highly correlated in their expression profile. Within each region, samples are also correlated with other samples from the same donor. (TIF) [file pcbi.1005064.s005.tif]

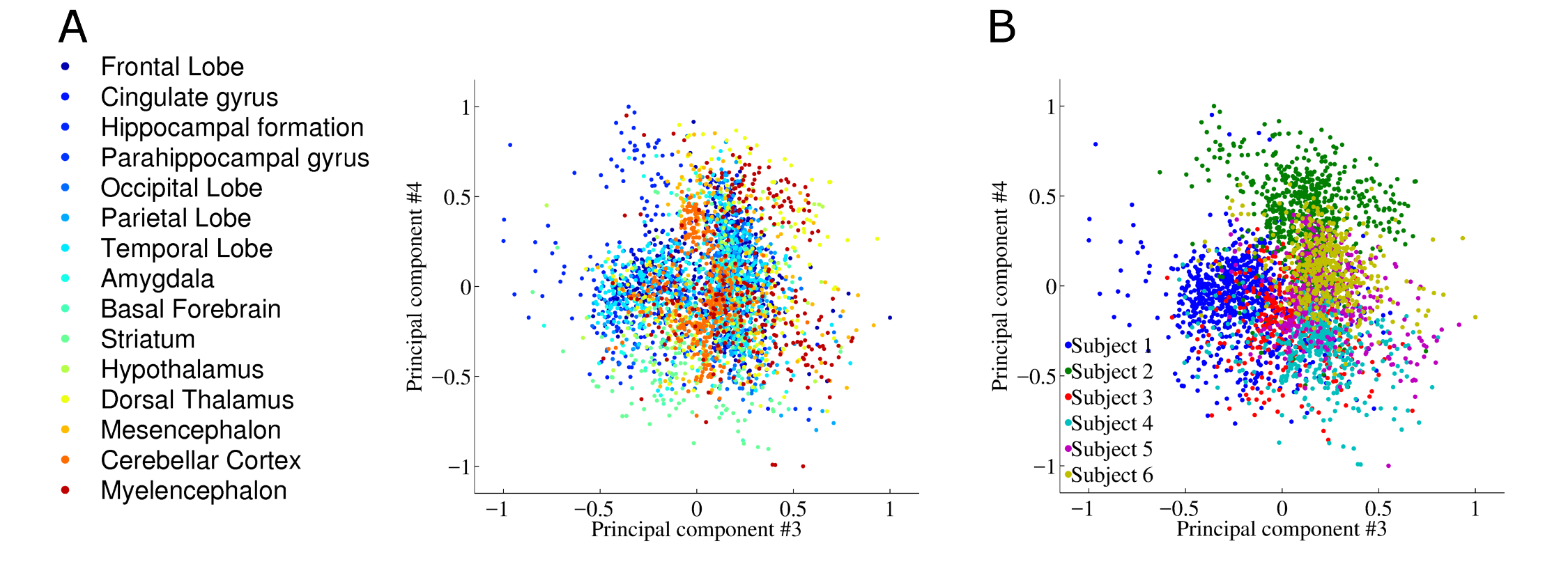

Supplement: S2 Fig — (A) Each point is a tissue sample. Samples are colored based on the position of the corresponding embryonic region. (B) Colors correpsond to donor identity. A significant fraction of the sample variance across the 3rd and 4th principal components is explained by subject-to-subject variablity. (TIF) [file pcbi.1005064.s006.tif]

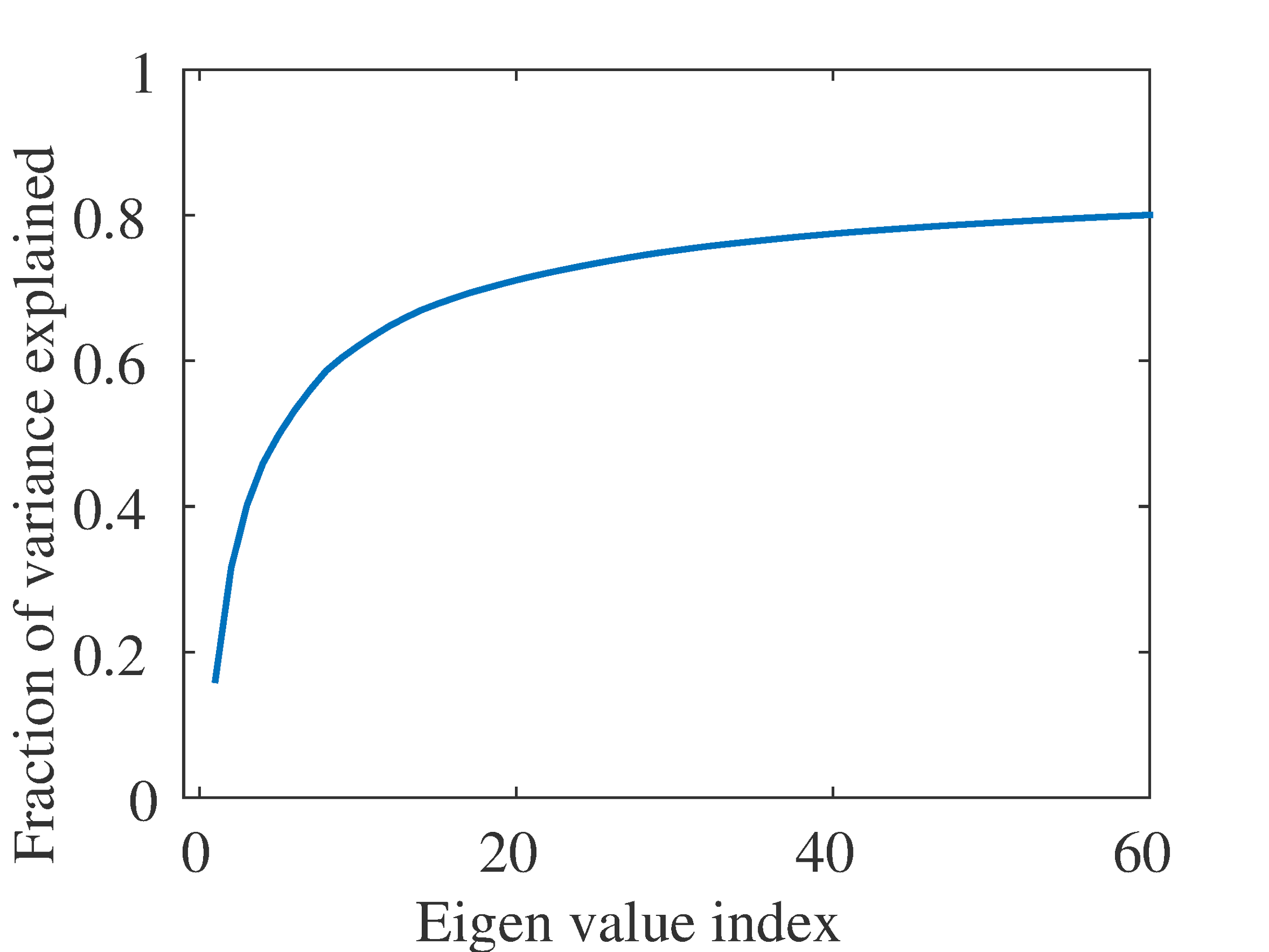

Supplement: S3 Fig — The first two principal components caputre 34% of the variance. Adding the 3rd and 4th principal components explain more than 51% of the sample-to-sample variance. (TIF) [file pcbi.1005064.s007.tif]

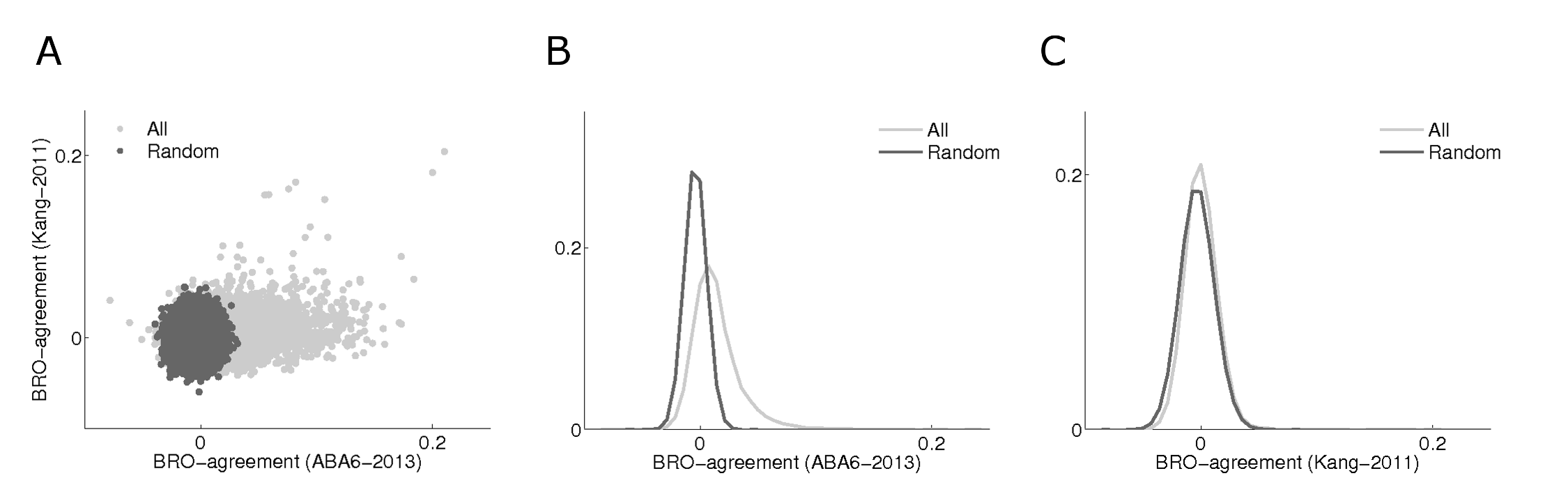

Supplement: S4 Fig — Color scheme and x-axis scale matche those of Fig 2. (A) A scatter plot showing BRO-agreement scores for the two datasets. Each light-grey dots corresponds to a single genes (a total of 17K genes). Dark-grey dots correspond to permuted data (see Methods). (B) Marginal distribution of BRO scores in the ABA6-2013 dataset. In the ABA6-2013 dataset, 11% of the genes (2207 out of 20773) are BRO-significant in the cortex. BRO score was also computed separately for each subject, using the ABA6-2013 dataset. With these per-subject scores, the number of BRO-significant genes varied considerably across the six subjects (30%, 33%, 5%, 37%, 16% and 18%), and the correlation of BRO scores between subjects is on average lower (mean Spearman Correlation of cortex BRO scores of a pair of subjects is 0.23 ± 0.13, compared with 0.76 ± 0.08 for the whole brain). (C) Marginal distribution of BRO scores in the Kang-2011 dataset. The large fraction of BRO-significant genes observed in ABA6-2013 was not found in the Kang-2011 dataset, where the two distributions largely overlap. (TIF) [file pcbi.1005064.s008.tif]

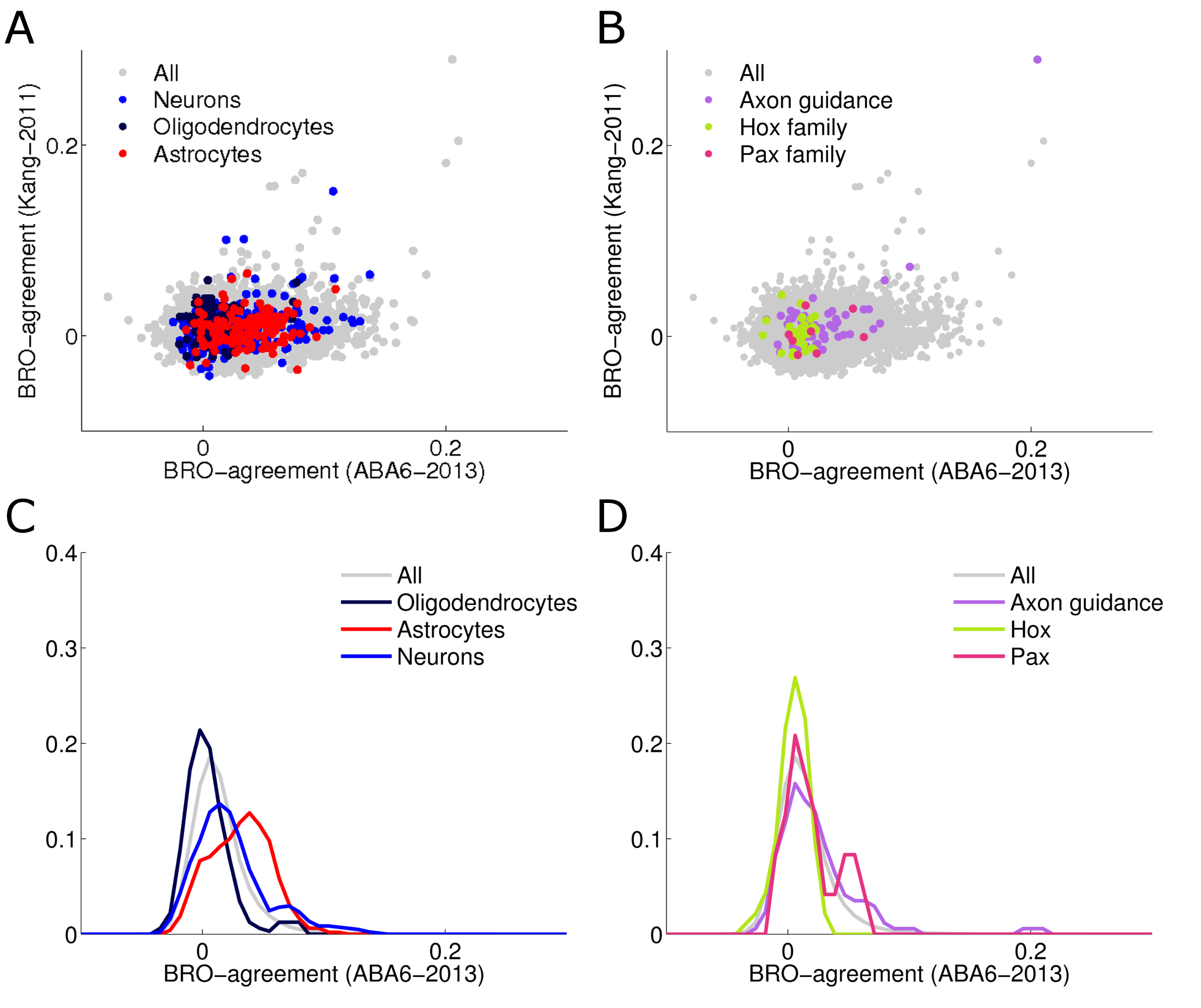

Supplement: S5 Fig — Color scheme and x-axis scale match those of Fig 3. (A, B) Neurons and astrocytes receive significantly higher agreement scores than all genes (Wilcoxon one tail test; neurons: p-value = 10-9, astrocytes: p-value = 10−22). Oligoodendrocyte genes are in less agreement with region-ontology than the full set of genes. Comparing these BRO scores to a randomized scores we find that 45% of the neuronal markers are cortex-BRO significant, 69% of the astrocytes markers are cortex-BRO significant and that 7% of the oligodendrocytes markers are cortex-BRO significant;). (C, D) Axon-guidance genes receive higher scores than genes on average (Wilcoxon one tail test, p-value = 10−3). Hox genes are less in agreement with region-ontology than the full set of genes (still significantly larger than random). PAX2 and PAX6 obtain high BRO scores. (TIF) [file pcbi.1005064.s009.tif]

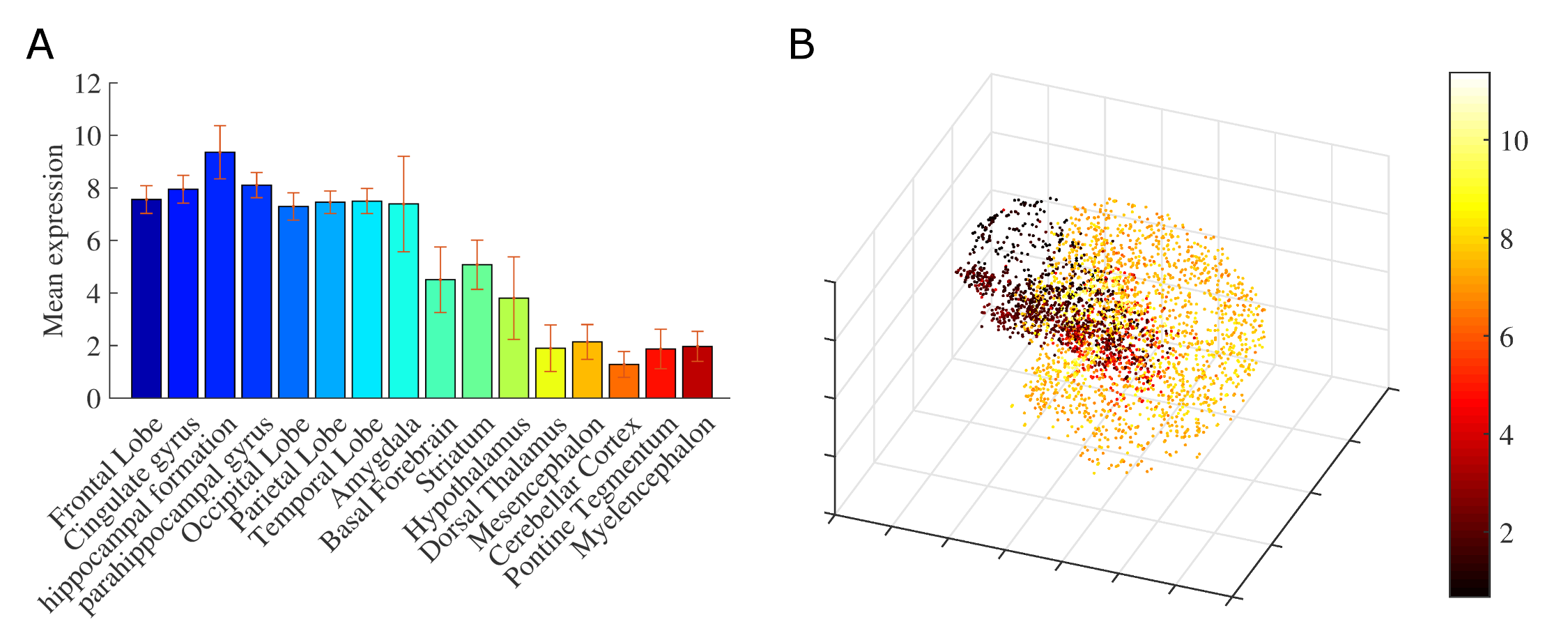

Supplement: S6 Fig — FEZF2 shows a clear transtion of its expression levels. The samples from the cortex show high experssion values where the samples midbrain has less and the samples of the hindbrain has the least expression. (A) The mean expression levels of the FEZF2 within different region in the human brain (the color scheme as consitant with that of Fig 1A. (B) We embed the samples in a 3D space using its MRI standartize location where the color of each sample shows the expression level of FEZF2. The scatter shows a transiation from high expression levels in the cortex to lower expression levels in the inner brain structures and to the hindbrain. (TIF) [file pcbi.1005064.s010.tif]

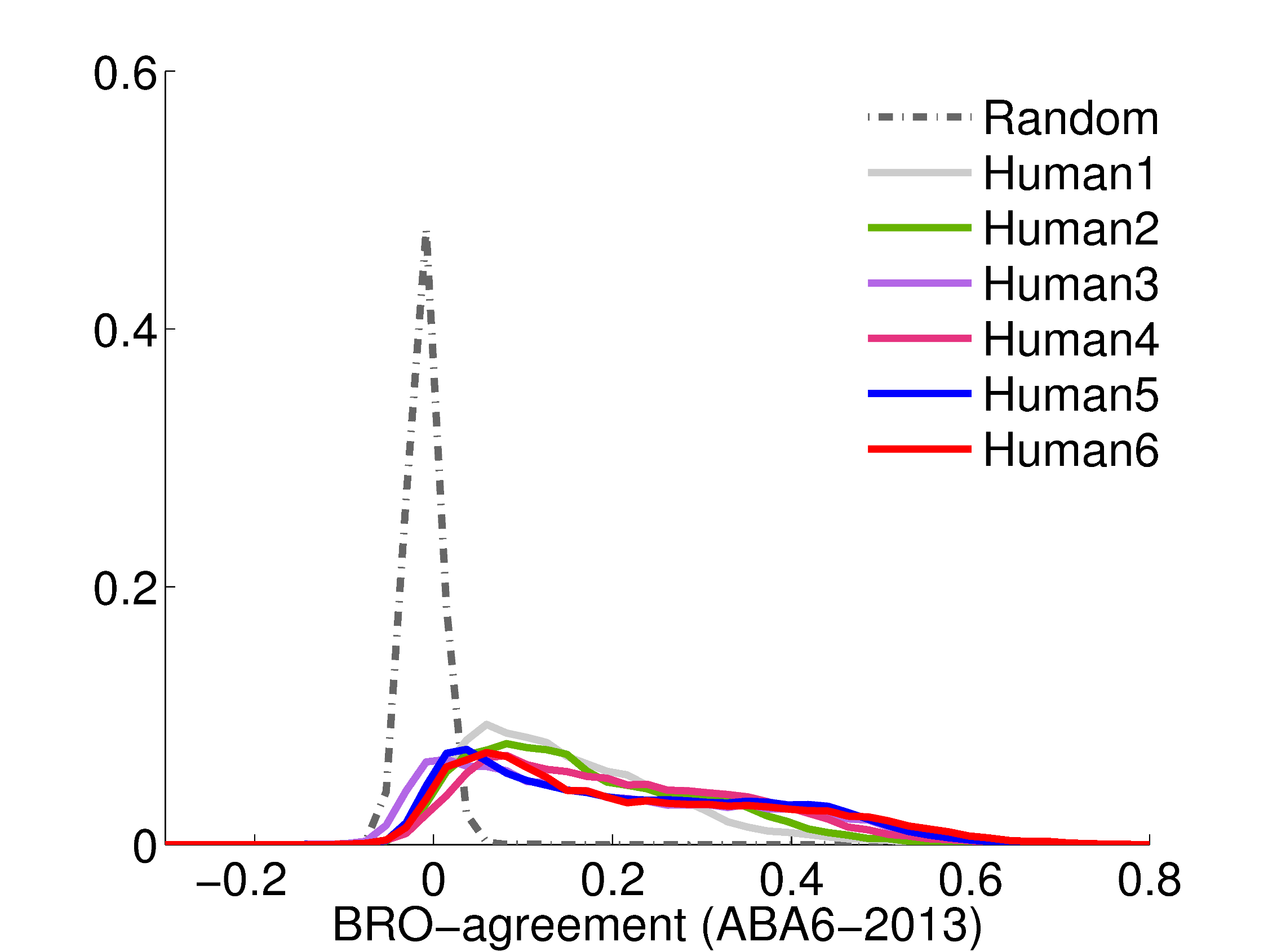

Supplement: S7 Fig — The percent of BRO-significant genes (p-value < 0.01) is stable when computed for each subject separately: 89%, 90%, 76%, 91%, 83% and 86%). (TIF) [file pcbi.1005064.s011.tif]

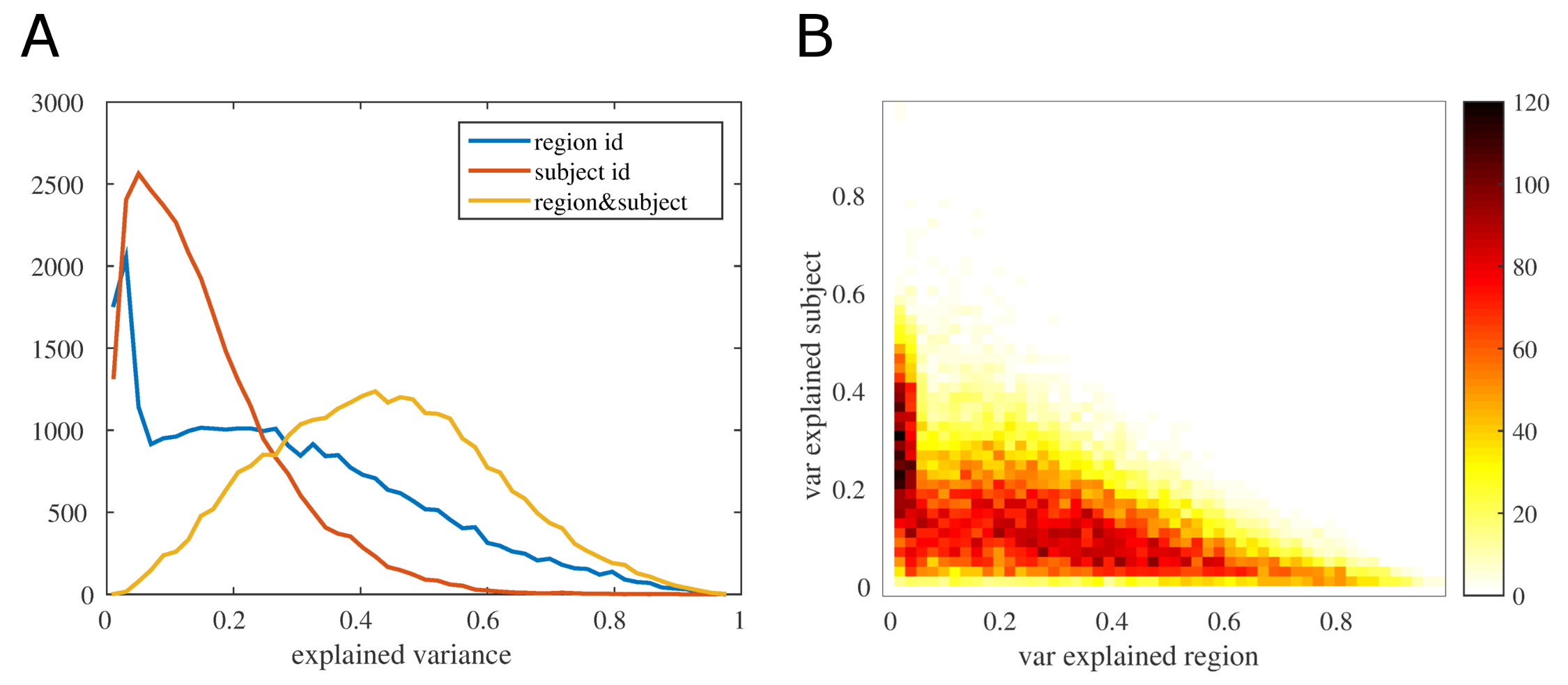

Supplement: S8 Fig — The joint information of subject id and region id explains almost half of the sample variance. For each gene, we represented the identity information as 1-hot-vectors, and computed the explained sample-variance by fitting a linear model (A) Distributions of explained variance across genes, as explained by region, subject or both. (B) The joint distribution of both the explained variance from region and from subject identity. (TIF) [file pcbi.1005064.s012.tif]

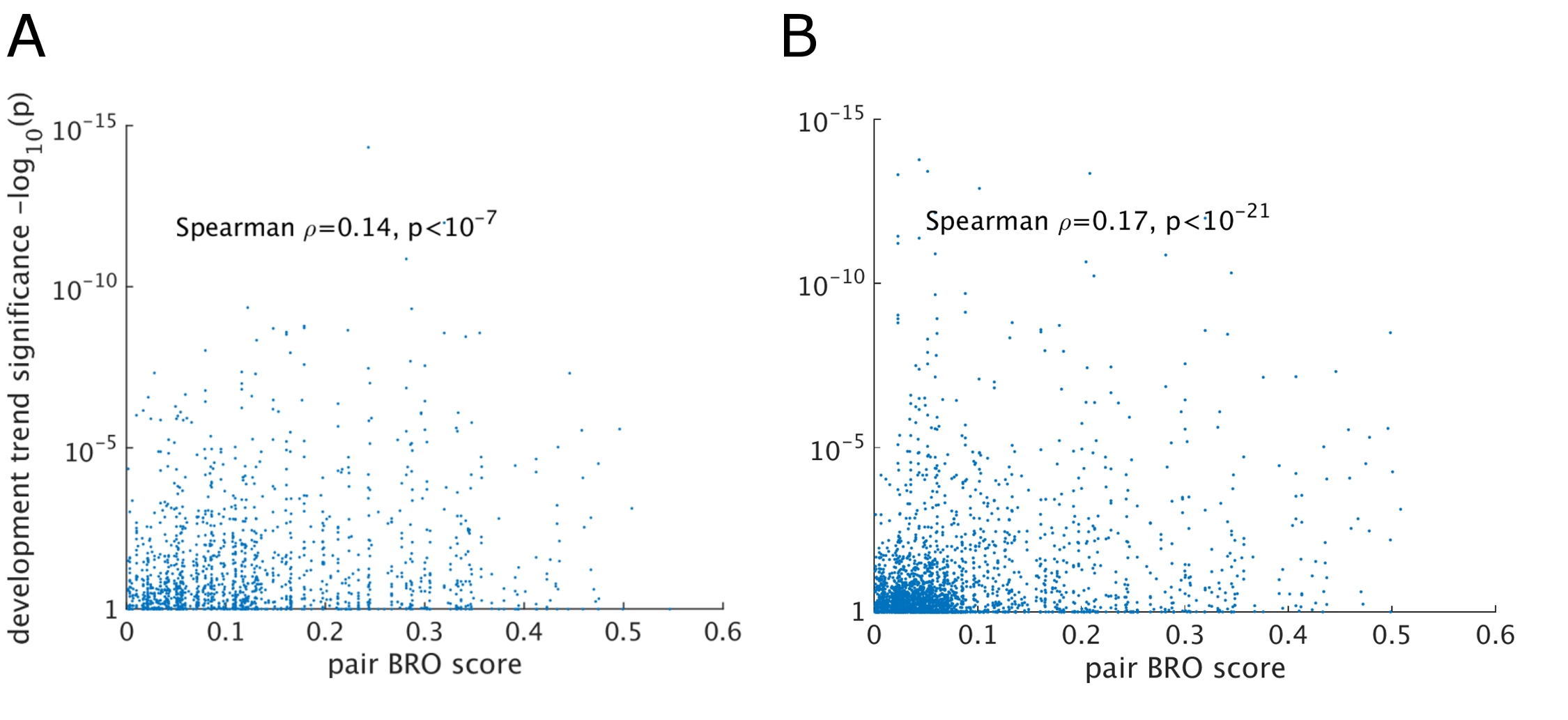

Supplement: S9 Fig — Trend significance of change in spatial correlation through life was quantified using the standard F-test comparing residual of a linear regression model with a constant model. Pair BRO score is the minimum BRO scores of the two genes in the pair. (A) We computed the developmental trend for each pair of genes from the KEGG-based set. We found that BRO-agreement scores are (weakly but significantly) positively correlated with having a significant trend. Each dot corresponds to one brain related sequence-similar pair. 17% of pairs (257/1496) had FDR-corrected significant (q<0.01) linear trend of their spatial correlation, as illustrated in Fig 6. (B) Similarly, we found that the developmental trend of paralog pairs which are brain related is also positively correlated with the BRO score. 6.8% of the brain related paralog-pairs show a significant trend (240/3503). (TIF) [file pcbi.1005064.s013.tif]

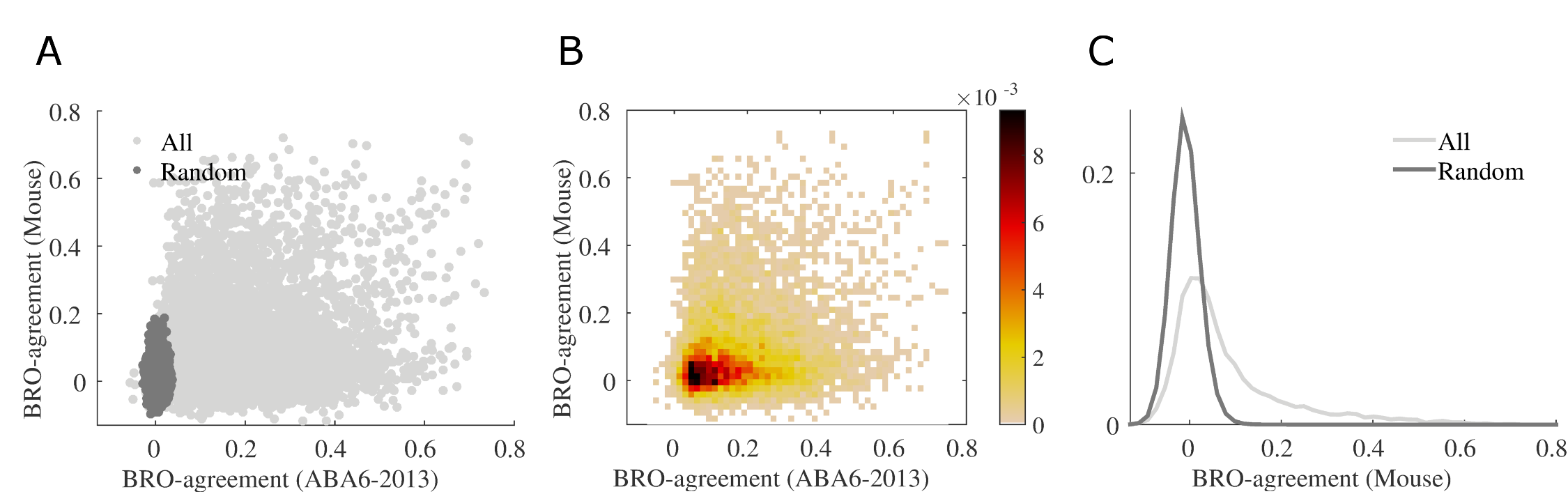

Supplement: S10 Fig — (A) 2D scatter of BRO scores for genes with a matching homolog in mouse (Zapala et al.[22]) and human (ABA2013) and a random baseline (B) 2D heat map of the joint distribution of BRO-scores for mouse and human. (C) The distribution of BRO-scores in mouse and a random baseline, showing that the fraction of BRO significant genes is smaller in the mouse dataset (30%) than in humans. (TIF) [file pcbi.1005064.s014.tif]

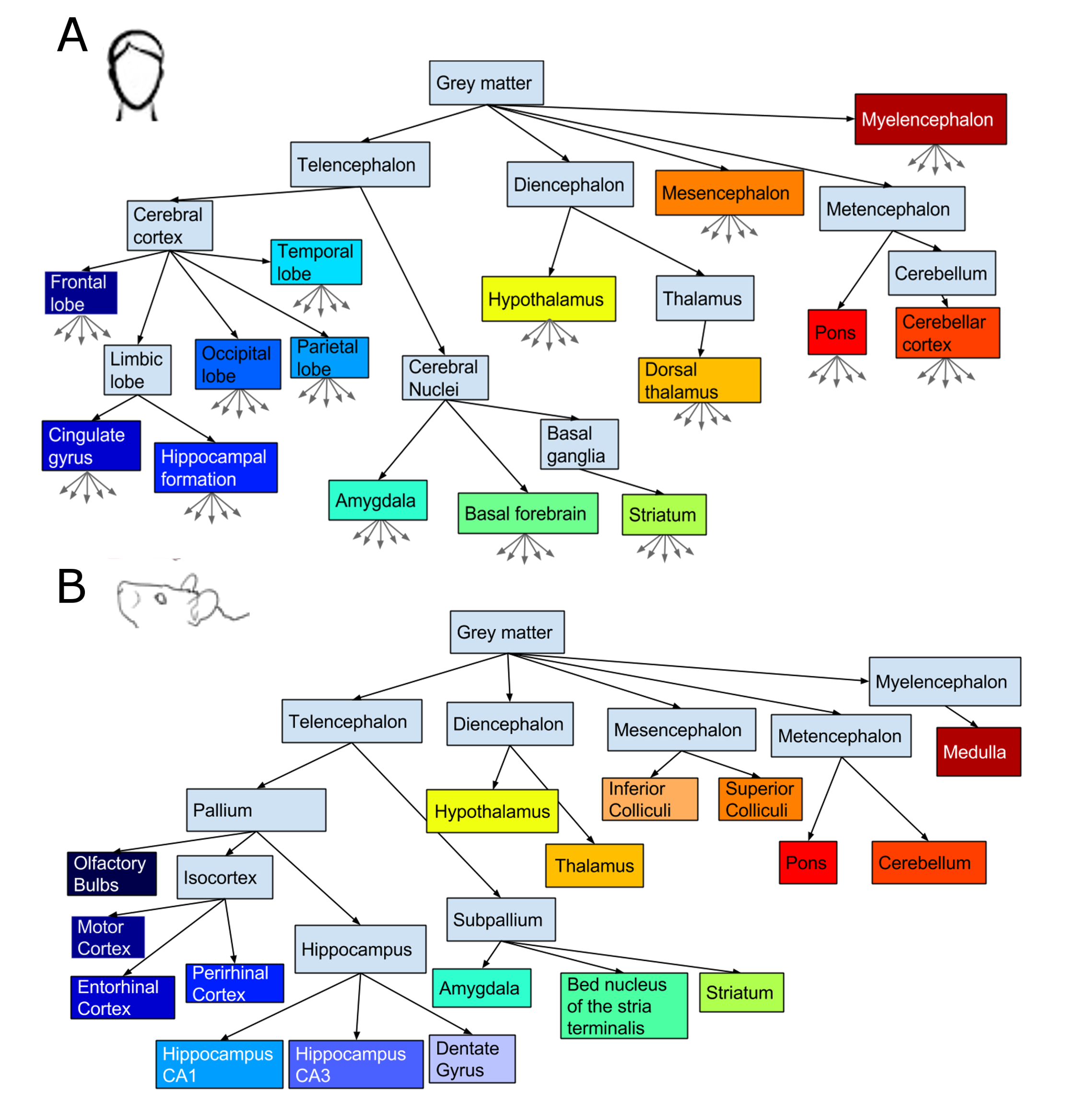

Supplement: S11 Fig — (A) The Allen ontology used for the human analysis. (B) The Allen ontology used for the mouse analysis. Both follow ontologies first devide the brain into the 5 embrionic vesiceles and then go into more detailed regionalization. The leaf regions are not the same since the data was gathered in different experiments each with a unique focus. (TIF) [file pcbi.1005064.s015.tif]
