# Supplementary material for: On Expression Patterns and Developmental Origin of Human Brain Regions
Source: PLoS Comput Biol. 2016 Aug 26;12(8):e1005064. doi: 10.1371/journal.pcbi.1005064 (PMC5001727; doi:10.1371/journal.pcbi.1005064)
Supplement: S1 Text — BRO in the human cortex and a comparsion between the BRO in human and in mouse. (DOCX) [file pcbi.1005064.s016.docx]

**Supplemental analysis**

## BRO in the human cortex.

Distinct expression patterns across major brain regions agree with the brain ontology for most genes. We further tested how expression differences are pronounced between sub regions of a single brain structure. Specifically, sub regions of the cortex share similar neuronal density, connectivity patterns and distribution of cell-types [4,25] and may perform interchangeable information processing tasks [26–29], raising the hypothesis that cortical subregions also share similar molecular profiles. Indeed, when clustering brain regions based on gene expression, cortical subregions group together [22], and when clustering brain voxels based on gene expression, cortical voxels cluster together [24]. On the other hand, cortical regions bear some distinct histological and molecular signatures, which form the basis of the classical division into regions [2]. We therefore set to characterize which genes show distinct areal expression patterns within the cortex, using the BRO index as computed over the cortical branches of the ontology tree. We find that a fairly large number of genes (2207 out of 20773, 11%) obtain significant BRO scores in the *ABA6-2013* dataset. This suggests that cortical sub-regions differ in their expression in a way that follows the cortical region ontology (S4 Figure). The fraction of significant genes was very low in Kang-2011 cortical data, presumably because the small number of samples was not sufficient to capture the lower variability across cortical regions. When considering which processes contribute to *subregional* variability, astrocyte-specific genes agree with the cortex region ontology significantly more than the average gene and more than other gene sets we tested (S5 Figure). This suggests that variability in astrocytes is a major determinant of the variability between cortical areas.

## BRO in the mouse brain.

We repeated BRO analysis on mouse brain data, to test the consistency of the results across species. The genome-wide expression in mice is highly related to the region ontology [22], and the per-gene BRO scores now allow us to estimate the effect at individual genes. We used expression data from Zapala et al. [22] and matched the 19 regions from their data to the Allen mouse brain region ontology (S11 Figure). **Many genes in the mouse also have a significant BRO score. However, it is a smaller fraction than that of the human data** (30% of the genes in the mouse are BRO-significant S10 Figure).
While these differences may reflect genuine cross-species differences of spatial expression patterns, they may also be attributed to various differences between the two datasets. First, the human and mouse ontologies share most of their structure in the upper part of the tree, but the leaf nodes in the ontologies differ, since each ontology matches regions from a different study. Also, the distribution of samples across regions differs between the mouse and the human datasets, with samples in mouse being distributed more uniformly across regions, and human cortical regions being more heavily sampled. It is hard to separate effects that reflect true cross species differences from differences that are due to the specific datasets used.
